# FFAR2 antagonizes TLR2- and TLR3-induced lung cancer progression via the inhibition of AMPK-TAK1 signaling axis for the activation of NF-κB

**DOI:** 10.1186/s13578-023-01038-y

**Published:** 2023-06-07

**Authors:** Mi-Jeong Kim, Ji Young Kim, Ji Hye Shin, Yeeun Kang, Ji Su Lee, Juhee Son, Soo-Kyung Jeong, Daesik Kim, Duk-Hwan Kim, Eunyoung Chun, Ki-Young Lee

**Affiliations:** 1 Department of Immunology, Samsung Biomedical Research Institute, Sungkyunkwan University School of Medicine, Suwon, 16419 Republic of Korea; 2 R&D Center, CHA Vaccine Institute, Seongnam-si, 13493 Republic of Korea; 3 Department of Precision Medicine, Sungkyunkwan University School of Medicine, Suwon, 16419 Republic of Korea; 4 Department of Molecular Cell Biology, Sungkyunkwan University School of Medicine, Suwon, 16419 Republic of Korea; 5 Department of Health Sciences and Technology, Samsung Advanced Institute for Health Sciences & Technology, Samsung Medical Center, Sungkyunkwan University, Seoul, 06351 Republic of Korea; 6 Single Cell Network Research Center, Sungkyunkwan University School of Medicine, Suwon, 16419 Republic of Korea

**Keywords:** FFAR2, SCFAs, Toll-like receptors, NF-κB, Lung cancer progression

## Abstract

**Background:**

Free fatty acid receptors (FFARs) and toll-like receptors (TLRs) recognize microbial metabolites and conserved microbial products, respectively, and are functionally implicated in inflammation and cancer. However, whether the crosstalk between FFARs and TLRs affects lung cancer progression has never been addressed.

**Methods:**

We analyzed the association between FFARs and TLRs using The Cancer Genome Atlas (TCGA) lung cancer data and our cohort of non-small cell lung cancer (NSCLC) patient data (n = 42), and gene set enrichment analysis (GSEA) was performed. For the functional analysis, we generated FFAR2-knockout (*FFAR2*KO) A549 and *FFAR2*KO H1299 human lung cancer cells and performed biochemical mechanistic studies and cancer progression assays, including migration, invasion, and colony-formation assays, in response to TLR stimulation.

**Results:**

The clinical TCGA data showed a significant down-regulation of FFAR2, but not FFAR1, FFAR3, and FFAR4, in lung cancer, and a negative correlation with TLR2 and TLR3. Notably, GSEA showed significant enrichment in gene sets related to the cancer module, the innate signaling pathway, and the cytokine-chemokine signaling pathway in FFAR2^Down^TLR2^Up^TLR3^Up^ lung tumor tissues (LTTs) vs. FFAR2^up^TLR2^Down^TLR3^Down^ LTTs. Functionally, treatment with propionate (an agonist of FFAR2) significantly inhibited human A549 or H1299 lung cancer migration, invasion, and colony formation induced by TLR2 or TLR3 through the attenuation of the cAMP-AMPK-TAK1 signaling axis for the activation of NF-κB. Moreover, *FFAR2*KO A549 and *FFAR2*KO H1299 human lung cancer cells showed marked increases in cell migration, invasion, and colony formation in response to TLR2 or TLR3 stimulation, accompanied by elevations in NF-κB activation, cAMP levels, and the production of C-C motif chemokine ligand (CCL)2, interleukin (IL)-6, and matrix metalloproteinase (MMP) 2 cytokines.

**Conclusion:**

Our results suggest that FFAR2 signaling antagonized TLR2- and TLR3-induced lung cancer progression via the suppression of the cAMP-AMPK-TAK1 signaling axis for the activation of NF-κB, and its agonist might be a potential therapeutic agent for the treatment of lung cancer.

**Supplementary Information:**

The online version contains supplementary material available at 10.1186/s13578-023-01038-y.

## Background

Lung cancer is the most frequent cause of cancer death worldwide and is influenced by numerous factors, including environmental exposure, genetics, diet, medications, prior disease/injury exposure, and the microbiome [1, 2]. Of them, the microbiome produces microbial metabolites, such as short-chain fatty acids (SCFAs), and conserved microbial products, such as pathogen-associated molecular patterns (PAMPs) [3, 4]. In the tumor microenvironment (TME), crosstalk between host immune cells or cancer and SCFAs or PAMPs is functionally diverse and complex, occurring through various signaling events to regulate tumor development and progression [5, 6]. Previous studies reported that SCFAs might be involved in hindering the pathological conditions of cancer and inflammatory bowel disease (IBD) due to their anti-inflammatory, immune-modulatory, and anti-neoplastic traits [7–9]. In contrast, PAMPs are functionally implicated in tumor formation and progression through the induction of chronic and persistent inflammation by the activation of pattern recognition receptors (PRRs), including toll-like receptors (TLRs), nucleotide oligomerization domain (NOD)-like receptors (NLRs), and retinoic acid-inducible gene (RIG-1)-like receptors (RLRs) [4, 10]. However, little is known about the mutual regulation between SCFAs and PAMPs for cancer progression.

SCFAs originate from microbiome fermentation or the diet [3]. SCFAs are recognized by free fatty acid receptors (FFARs), which are composed of FFAR1, FFAR2, FFAR3, and FFAR4 [3, 11]. SCFAs, such as acetate (an FFAR2 ligand), propionate (an FFAR2 and FFAR3 ligand), and butyrate (an FFAR3 ligand), have been shown to suppress inflammation and cancer [12, 13], and are thereby considered potential agents for cancer intervention [6, 14, 15]. Accumulating recent evidence indicated that SCFAs were able to inhibit cancer invasion, migration, and proliferation in several cancers, such as colon, fibrosarcoma, and prostate cancer, by modulating cell survival and mobility for cancer progression [16–18]. Besides SCFAs, the microbiome produces PAMPs and microbe-associated molecular patterns (MAMPs), which are small molecular motifs conserved within a class of microbes and functionally implicated in cancer progression through TLR-mediated signals [4, 19, 20]. In terms of pathophysiology, chronic or persistent inflammation has been considered a high-risk factor for lung cancer disease by promoting cancer development and progression [21]. Recently, it was demonstrated that TLRs could cause tumor development and progression by orchestrating cellular signaling pathways, such as nuclear factor (NF)-κB signaling, Src/MAPK signaling, Wnt signaling, and phosphoinositide-3 kinase (PI3K)/Akt signaling [22–26]. Importantly, it has been reported that TLR expression in non-small-cell lung carcinoma (NSCLC) was markedly higher than in normal lung tissue [27–29]. Consequently, the identification of novel regulators capable of inhibiting TLR signals might provide novel insight into the development of therapeutic targets for the treatment of lung cancer.

Herein, we explored the functional association between FFARs and TLRs for lung cancer progression. We found that FFAR2 was negatively associated with TLR2 and TLR3 in lung cancer. Propionate, an agonist of FFAR2, antagonized TLR2- and TLR3-induced lung cancer migration, invasion, and colony formation by inhibiting the AMPK-TAK1 signaling axis for the activation of NF-κB. Notably, FFAR2-knockout (*FFAR2*KO) A549 and *FFAR2*KO H1299 lung cancers exhibited enhanced cancer progression induced by TLR2 or TLR3 stimulation, accompanied by increases in NF-κB, cAMP levels, and the production of C-C motif chemokine ligand (CCL)2, interleukin (IL)-6, and matrix metalloproteinase (MMP)2 cytokines. Collectively, these results suggest that FFAR2 signaling functionally antagonizes lung cancer progression induced by TLR2 or TLR3 via the suppression of the AMPK-TAK1 signaling axis for the activation of NF-κB.

.

## Methods

### NSCLC patients, tumors, and matched normal specimens

Lung tumor tissue (LTT) and matched lung normal tissue (mLNT) of NSCLC patients (n = 42) were obtained from the Samsung Medical Center (SMC, Seoul, Korea) during surgery in accordance with the ethical principles stated in the Declaration of Helsinki. Lung tumors and matched normal specimens of the enrolled patients were immediately frozen in liquid nitrogen and stored at -80 °C until use. LTT and mLNT were verified by pathologists (SMC, Department of Pathology, Seoul, Korea). This study was conducted in accordance with the ethical principles stated in the Declaration of Helsinki and approved by the Institutional Review Board (IRB#: 2010-07-204) of the Samsung Medical Center. Written informed consent to use pathological specimens for research was obtained from all patients prior to surgery.

### Cells

A549 cells (human lung cancer cell line; ATCC, CCL-185) and H1299 cells (human non-small cell lung cancer cell line; ATCC, CRL-5803) were maintained in RPMI 1640 medium (Sigma Aldrich, 31800-022) supplemented with 10% fetal bovine serum (FBS), penicillin (100 µg/mL), and streptomycin (100 µg/mL) in a 5% CO_2_ humidified atmosphere at 37 °C.

### Generation of ***FFAR2***-knockout (***FFAR2*** KO) cell line using CRISPR/Cas9

To generate *FFAR2*KO lung cancer cells using the CRISPR/Cas9 gene editing method, we used two vector systems, sgRNA and cas9 vectors, as previously described [30]. The sgRNA and cas9 vectors were kindly provided by Dr. Daesik Kim (Sungkyunkwan University School of Medicine, Suwon, Korea). Briefly, *FFAR2*-guide RNA sequences for CRISPR/Cas9 were designed on the CRISPR design website (http://crispr.mit.edu/) provided by the Feng Zhang laboratory. The guide RNA sequences for *FFAR2* were gRNA1, 5’-CACCGGTCTGCGCCCTCACGAGTTT-3’ and 3’-CCAGACGCGGGAGTGCTCAAACAAA-5’; gRNA2, 5’-CACCG TGCAGTACAAGCTCTCCCGC-3’ and 3’-CACGTCATGTTCGAGAGGGCGCAAA-5’; gRNA3, 5’-CACCGCACCGATAACCAGTTGGACG-3’ and 3’-CGTGGCTATTGGTCAACCTGCCAAA-5’. Complementary oligonucleotides to guide RNAs (gRNAs) were annealed and cloned into a sgRNA vector. The sgRNA vector expressing FFAR2 gRNA and the cas9 vector expressing cas9 were transfected into A549 or H1299 cells using Lipofectamine 2000, according to the manufacturer’s instructions. After two weeks, colonies were isolated into 96-well plates, and the expression levels of FFAR2 were analyzed by Western blots.

### Antibodies and reagents

Anti-phospho-AMPKα (Thr172, 2531), anti-AMPKα (2532), anti-phospho-TAK1 (Ser412, 9339), anti-TAK1 (4505), anti-phospho-NF-κB p65 (Ser536, 93H1, 3033), and anti-NF-κB p65 (D14E12, 8242) antibodies were purchased from Cell Signaling Technology (Danvers, MA, USA). Anti-GPCR GPR43 (FFAR2, ab131003) antibody was purchased from Abcam (Cambridge, MA, USA). Goat anti-rabbit IgG antibody (horseradish peroxidase (HRP)-labeled) (GTX213110-01) was purchased from GeneTex Inc. (Irvine, CA, USA). Dimethyl sulfoxide (DMSO; 472,301), paraformaldehyde (P6148), Triton X-100 (T8787), gentamicin (G1272), deoxycholate (D6750), and Dulbecco’s phosphate-buffered saline (DPBS; D8537) were purchased from Sigma-Aldrich (St Louis, MO, USA). Heat-killed *Listeria monocytogenes* (HKLM, tlrl-hklm) and polyinosinic-polycytidylic acid (poly(I:C), tlrl-pic) were purchased from InvivoGen (San Diego, CA, USA). Lipofectamine 2000 (11,668,019) was purchased from Thermo Fisher Scientific (Waltham, MA, USA).

### Western blotting

Western blotting (WB) was performed as previously described [31–41]. Briefly, A549 or H1299 lung cancer cells (5 × 10^5^ cells per well) were seeded into 6-well plates and cultured (80 ~ 90% confluent). Cells were treated with vehicle (DMSO, 0.1% v/v concentration), HKLM (2 × 10^8^ cells/mL), and poly(I:C) (20 µg/mL) in the presence or absence of propionate (1 mM) for 6 h. After collecting the cells, cell lysates were separated by sodium dodecyl sulfate-polyacrylamide gel electrophoresis (SDS-PAGE, 8 ~ 12%) and immune-probed with anti-pho-AMPK, anti-pho-TAK1, anti-pho-p65, anti-p65, and anti-GAPDH antibodies (as the loading control). Ctrl A549, *FFAR2*KO A549, Ctrl H1299, and *FFAR2*KO H1299 cells (5 × 10^5^ cells per well) were seeded into 6-well plates and cultured (80 ~ 90% confluent). Cells were treated with vehicle (DMSO, 0.1% v/v concentration), HKLM (2 × 10^8^ cells/mL), and poly(I:C) (20 µg/mL) for 6 h. Cell lysates were separated by SDS-PAGE (8 ~ 12%) and immune-probed with anti-pho-AMPK, anti-pho-TAK1, and anti-GAPDH antibodies (as the loading control).

### NF-κB luciferase reporter assay

The luciferase reporter assay was performed as previously described [38]. Briefly, A549 and H1299 lung cancer cells were seeded into 24-well tissue culture plates to get a 40–60% confluence 24 h later. Cells were transfected with pBIIx-luc NF-κB-dependent reporter construct and the Renilla luciferase vector (Promega, Madison, WI, USA). At 24 h post-transfection, the cells were treated with vehicle (DMSO, 0.1% v/v concentration), HKLM (2 × 10^8^ cells/mL), and poly(I:C) (25 µg/mL) in the presence or absence of different concentrations of propionate for 24 h. The cells were lysed, and luciferase activity was measured using a dual luciferase assay kit (Promega). Ctrl A549, *FFAR2*KO A549, Ctrl H1299, and *FFAR2*KO H1299 cells were transfected with pBIIx-luc NF-κB-dependent reporter construct and the Renilla luciferase vector (Promega). At 24 h post-transfection, the cells were treated with vehicle (DMSO, 0.1% v/v concentration), HKLM (10^8^ cells/mL), and poly(I:C) (20 µg/mL) in the presence or absence of propionate (1 mM) for 6 h. The cells were lysed, and luciferase activity was measured using a dual luciferase assay kit (Promega). The luciferase assay was carried out in triplicate in at least three independent experiments.

### Measurement of cAMP levels

cAMP production was measured as previously described [42]. Briefly, A549 and H1299 lung cancer cells (4 × 10^4^ cells/well) were treated with vehicle (DMSO, 0.1% v/v concentration), HKLM (10^8^ cells/mL), and poly(I:C) (20 µg/mL) in the presence or absence of propionate (1 mM) for 36 h. cAMP levels were quantified using a cAMP Alpha ELISA kit (#AL312, PerkinElmer Inc, MA, USA) following the manufacturer’s instructions. Ctrl A549, *FFAR2*KO A549, Ctrl H1299, and *FFAR2*KO H1299 cells (4 × 10^4^ cells/well) were treated with vehicle (DMSO, 0.1% v/v concentration), HKLM (10^8^ cells/mL), and poly(I:C) (20 µg/mL) for 36 h. cAMP levels were quantified using a cAMP AlphaLISA kit (PerkinElmer, #AL312) following the manufacturer’s instructions.

### Measurement of CCL2, IL-6, and MMP2 cytokines

The production of CCL2, IL-6, and MMP2 cytokines was measured as previously described [20]. Briefly, A549 and H1299 lung cancer cells (4 × 10^4^ cells per well /96-well plate) were treated with vehicle (DMSO, 0.1% v/v concentration), HKLM (10^8^ cells/mL), and poly(I:C) (20 µg/mL) in the presence or absence of propionate (1 mM) for 24 h. CCL2 (DCP00), IL-6 (D6050), and MMP2 (DMP2F0) levels in the supernatant fractions were measured by ELISA (R&D Systems, Minneapolis, MN, USA) according to the manufacturer’s protocols. Ctrl A549, *FFAR2*KO A549, Ctrl H1299, and *FFAR2*KO H1299 cells (4 × 10^4^ cells per well /96-well plate) were treated with vehicle (DMSO, 0.1% v/v concentration), HKLM (10^8^ cells/mL), and poly(I:C) (20 µg/mL) for 24 h. CCL2 (DCP00), IL-6 (D6050), and MMP2 (DMP2F0) levels in the supernatant fractions were measured by ELISA (R&D Systems) according to the manufacturer’s protocols.

### Wound-healing migration assay

A wound-healing migration assay was performed following previous protocols [31, 34–36]. Briefly, A549 and H1299 lung cancer cells were seeded into 12-well plates and cultured to reach about 90% confluence. Cell monolayers were gently scratched with a sterile pipet tip and washed with a culture medium. After removing floating cells and debris, the cells were treated with vehicle (DMSO, 0.1% v/v concentration), HKLM (10^8^ cells/mL), and poly(I:C) (20 µg/mL) in the presence or absence of propionate (1 mM). Cell images were captured after culturing for different periods. Gap width was measured using ImageJ software and the results were expressed in % of wound size considering the time. Ctrl A549, *FFAR2*KO A549, Ctrl H1299, and *FFAR2*KO H1299 cells were seeded into 12-well plates and cultured to reach about 90% confluence. Cell monolayers were gently scratched with a sterile pipet tip and washed with a culture medium. After removing floating cells and debris, the cells were treated with vehicle (DMSO, 0.1% v/v concentration), HKLM (10^8^ cells/mL), and poly(I:C) (20 µg/mL). Cell images were captured after culturing for different periods.

### Transwell invasion assay

The Transwell invasion assay was performed following previous protocols [31, 34–36]. Briefly, A549 and H1299 lung cancer cells (1–2 × 10^4^) were suspended in a culture medium (200 µL) and added to the upper compartment of a 24-well Transwell® chamber containing a polycarbonate filter with 8-µm pores and coated with 60 mL of Matrigel (Sigma Aldrich, E1270; 1:9 dilution). The cells were treated with vehicle (DMSO, 0.1% v/v concentration), HKLM (10^8^ cells/mL), and poly(I:C) (20 µg/mL) in the presence or absence of propionate (1 mM) for 24 h. The invaded cells were stained with 0.5% crystal violet (Sigma-Aldrich, C6158-50G) and quantified by counting the number of cells. Ctrl A549, *FFAR2*KO A549, Ctrl H1299, and *FFAR2*KO H1299 cells (1–2 × 10^4^) were suspended in a culture medium (200 µL) and added to the upper compartment of a 24-well Transwell® chamber containing a polycarbonate filter with 8-µm pores and coated with 60 mL of Matrigel (Sigma Aldrich, E1270; 1:9 dilution). The cells were treated with vehicle (DMSO, 0.1% v/v concentration), HKLM (10^8^ cells/mL), and poly(I:C) (20 µg/mL) for 24 h. The invaded cells were stained with 0.5% crystal violet (Sigma-Aldrich, C6158-50G) and quantified by counting the number of cells.

### Anchorage-independent soft agar colony-formation assay

The anchorage-independent soft agar colony-formation assay was performed following previous protocols [36, 43]. Briefly, A549 and H1299 lung cancer cells (1 × 10^4^ cells per well) mixed with 0.3% Difco Noble Agar (BD Biosciences, CA, USA) in a complete medium were plated on the bottom of the 0.5% agar layer in a 6-well plate with complete medium. Culture medium (1.5 mL) with vehicle (DMSO, 0.1% v/v concentration), HKLM (2 × 10^8^ cells/mL), and poly(I:C) (20 µg/mL) in the presence or absence of propionate (1 mM) was added on top of the layer and the cells were incubated at 37 °C for 4 weeks. Ctrl A549, *FFAR2*KO A549, Ctrl H1299, and *FFAR2*KO H1299 cells (1 × 10^4^ cells per well) mixed with 0.3% Difco Noble Agar (BD Biosciences) in a complete medium were plated on the bottom of the 0.5% agar layer in a 6-well plate with complete medium. Culture medium (1.5 mL) with vehicle (DMSO, 0.1% v/v concentration), HKLM (2 × 10^8^ cells/mL), and poly(I:C) (20 µg/mL) were added on top of the layer, and the cells were incubated at 37 °C for 4 weeks.

### Microarray analysis

Microarray analysis was performed as previously described [32, 44, 45]. Briefly, total RNA was extracted from the LTT and matched normal tissues of 42 patients with NSCLC using Trizol (Thermo Fisher Scientific, 15,596,026) and purified using RNeasy columns (74,106, Qiagen, Hilden, Germany) according to each manufacturer’s protocol. We analyzed mRNA expression using HumanHT-12 expression BeadChips (Illumina, San Diego, CA, USA). The microarray data were pre-processed for background adjustment and normalization using the Bioconductor Lumi package (https://bioconductor.org/biocLite.R).

### Gene set enrichment analysis (GSEA)

The different magnitudes (Mags) of FFAR2, TLR2, and TLR3 expression were obtained from the pre-processed microarray data between LTT (n = 42) and matched LNT (n = 42). Six NSCLC LTT samples (Group A, 3 LTTs with FFAR2^Down^TLR2^Up^TLR3^Up^; Group B, 3 LTTs with FFAR2^Up^TLR2^Down^TLR3^Down^) were selected. Significant differences between Group A and Group B were analyzed by GSEA (http://www.gsea-msigdb.org/gsea/index.jsp) [46].

### Statistical analysis

All in vitro data are expressed as the mean ± SD of triplicate samples. Statistical significances were analyzed by ANOVA or the Student’s t-test using GraphPad Prism 5.0 (GraphPad Software, San Diego, CA, USA). The values represent the mean ± SD of three independent experiments.

## Results

### FFAR2 is negatively correlated with the expression of TLR2/3 in lung cancer

**Fig. 1:**
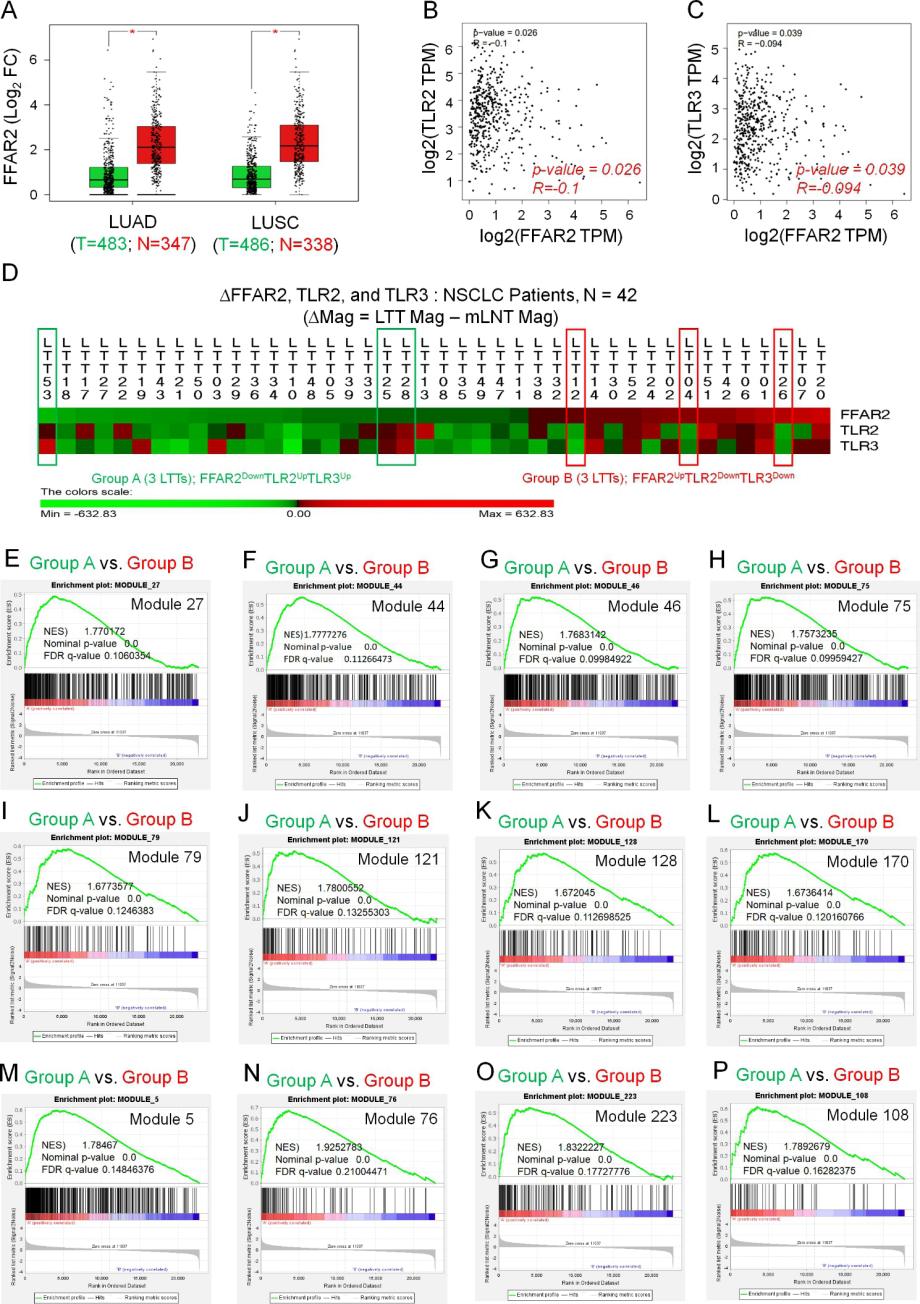
The association between FFARs and TLRs in lung cancer. **A**. FFAR2 was significantly down-regulated in LUAD and LUSC. FFAR2 expression was compared between tumor and normal tissues by gene expression profiling interactive analysis (GEPIA, http://gepia.cancer-pku.cn/detail.php?gene=FFAR2) data. *P < 0.05. **B** and **C**. FFAR2 expression was negatively correlated with TLR2 and TLR3 expression in LUAD (**B**, TLR2, p-value = 0.026, R = -0.1; **C**, TLR3, p-value = 0.039, R = -0.094). **D**. The different magnitudes of FFAR2 (∆FFAR2), TLR2 (∆TLR2), and TLR3 (∆TLR3) were analyzed in NSCLCs (n = 42, LTT vs. matched LNT). **E-P**. Based on ∆FFAR2, ∆TLR2, and ∆TLR3, six LTTs were selected (Group A, three LTTs with FFAR2^Down^TLR2^Up^TLR3^Up^, indicated by green boxes in **D**; Group B, three LTTs with FFAR2^Up^TLR2^Down^TLR3^Down^, indicated by red boxes in **D**). GSEA (http://www.gsea-msigdb.org/gsea/index.jsp) was performed for Group A vs. Group B. Twelve gene sets for cancer modules were significantly enriched in Group A vs. Group B. NES and the nominal p-value are indicated in each inner panel

To obtain insight into the association of FFARs and TLRs in lung cancer, the expression of FFARs was evaluated by gene expression profiling interactive analysis data (GEPIA, http://gepia.cancer-pku.cn). Of the four FFARs, FFAR2 expression was significantly decreased in lung adenocarcinoma (LUAD) and lung squamous cell carcinoma (LUSC) (Fig. 1A, left, LUAD; right, LUSC: *P < 0.05), whereas no significant changes were observed in the other three FFARs, including FFAR1, FFAR3, and FFAR4 (Supplementary Figure S1A, FFAR1; Figure S1B, FFAR3; Figure S1C, FFAR4). Correlation analysis between FFAR2 and TLRs in LUAD revealed that FFAR2 expression was negatively correlated with TLR2 and TLR3 (Fig. 1B, TLR2, p-value = 0.026, R = -0.1; Fig. 1C, TLR3, p-value = 0.039, R = -0.094), but not with TLR1, TLR4, TLR6, TLR7, TLR8, or TLR9 (Supplementary Figures S2A-S2F). To verify the results, we utilized the microarray data of NSCLC patients (n = 42) and compared the different magnitude in ∆FFAR2, ∆TLR2, and ∆TLR3 expression between LTT samples and matched LNT samples (Fig. 1D and Supplementary Table S1). To determine whether the reverse correlation between FFAR2 and TLR2/3 was associated with gene sets in the cancer module, we selected six NSCLC LTT samples (Fig. 1D: Group A, 3 LTTs with FFAR2^Down^TLR2^Up^TLR3^Up^; Group B, 3 LTTs with FFAR2^Up^TLR2^Down^TLR3^Down^) and performed GSEA (https://www.gsea-msigdb.org) between the Group A LTTs and the Group B LTTs. Fourteen gene sets related to the cancer module were significantly enriched in Group A LTTs with FFAR2^Down^TLR2^Up^TLR3^Up^ vs. Group B LTTs with FFAR2^Up^TLR2^Down^TLR3^Down^. (Fig. 1E-P and Supplementary Figures S3A-S3B). These results suggest that FFAR2^Down^TLR2^Up^TLR3^Up^ NSCLC is positively associated with cancer modules compared to FFAR2^Up^TLR2^Down^TLR3^Down^ NSCLC.

### Gene sets for innate and cytokines signals are significantly enriched in FFAR2^Down^TLR2^Up^TLR3^Up^ NSCLC vs. FFAR2^Up^TLR2^Down^TLR3^Down^NSCLC

**Fig. 2:**
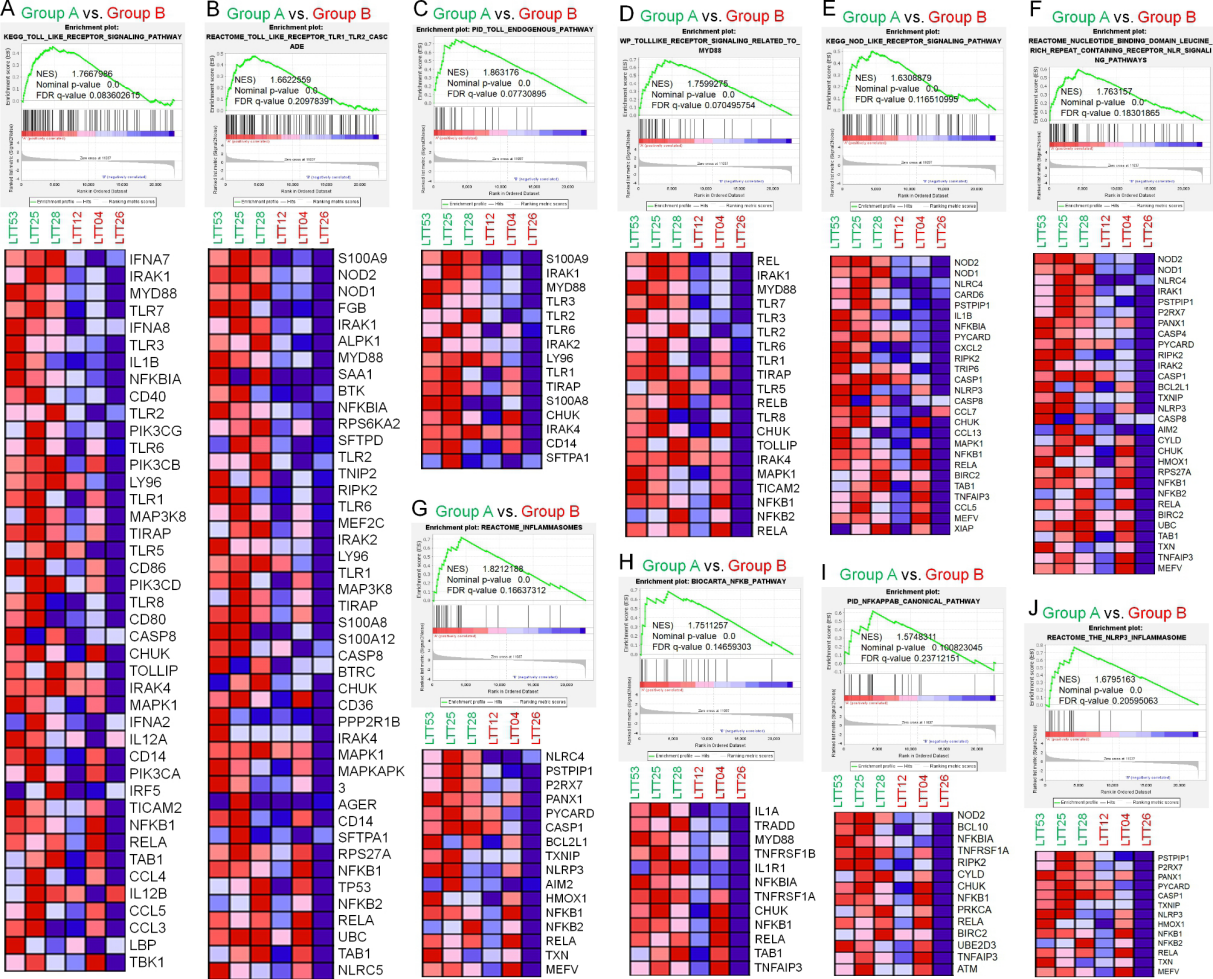
Gene sets related to innate signals were enriched in FFAR2^Down^TLR2^Up^TLR3^Up^ LTTs vs. FFAR2^Up^TLR2^Down^TLR3^Down^ LTTs. **A-J**. GSEA (http://www.gsea-msigdb.org/gsea/index.jsp) was performed for Group A (FFAR2^Down^TLR2^Up^TLR3^Up^ LTTs) vs. Group B (FFAR2^Up^TLR2^Down^TLR3^Down^ LTTs). Ten gene sets related to innate signals were significantly enriched in FFAR2^Down^TLR2^Up^TLR3^Up^ LTTs vs. FFAR2^Up^TLR2^Down^TLR3^Down^ LTTs (**A**. toll-like receptor signaling pathway; **B**. TLR1/2 cascade; **C**. toll endogenous pathway; **D**. TLR signaling related to MyD88; **E** and **F**. NOD and NLR signaling pathways; **G**. inflammasomes; **H**. NF-κB pathway; **I**. NF-κB canonical pathway; **J**. NLRP3 in inflammasome). NES and the nominal p-value are indicated in each inner panel

To determine whether the reverse correlation between FFAR2 and TLR2/3 in NSCLC patients was associated with gene sets for FFAR2- or TLR2/3-related pathways, GSEA was further performed in FFAR2^Down^TLR2^Up^TLR3^Up^ NSCLC samples (Group A) vs. FFAR2^Up^TLR2^Down^TLR3^Down^ NSCLC samples (Group B). Importantly, gene sets related to the toll-like receptor signaling pathway (Fig. 2A), TLR1/2 cascade (Fig. 2B), toll endogenous pathway (Fig. 2C), TLR signaling related to MyD88 (Fig. 2D), NOD, and NLR signaling pathways (Fig. 2E, F) were significantly enriched in FFAR2^Down^TLR2^Up^TLR3^Up^ NSCLC (Group A) vs. FFAR2^Up^TLR2^Down^TLR3^Down^ NSCLC (Group B). In addition, gene sets for inflammasomes (Fig. 2G), the NF-κB pathway (Fig. 2H), NF-κB canonical pathway (Fig. 2I), and NLRP3 in inflammasome (Fig. 2J) were also enriched in FFAR2^Down^TLR2^Up^TLR3^Up^ NSCLC (Group A) vs. FFAR2^Up^TLR2^Down^TLR3^Down^ NSCLC (Group B). These results suggest that FFAR2 down-regulation is positively associated with pattern recognition receptor (PRRs)-mediated signaling pathways.

**Fig. 3:**
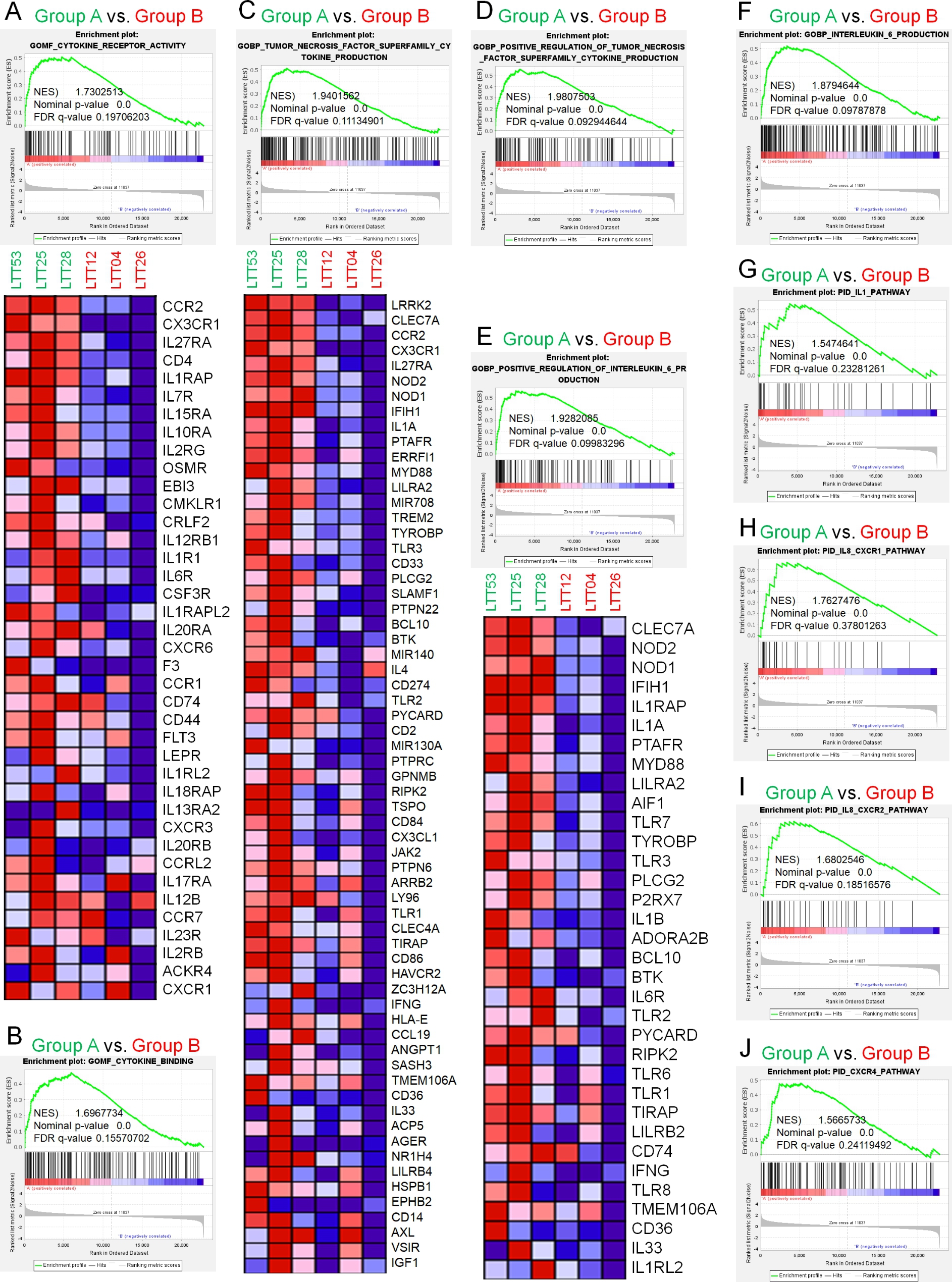
Gene sets related to cytokines and chemokines were enriched in FFAR2^Down^TLR2^Up^TLR3^Up^ LTTs vs. FFAR2^Up^TLR2^Down^TLR3^Down^ LTTs. **A-J**. GSEA (http://www.gsea-msigdb.org/gsea/index.jsp) was performed for Group A (FFAR2^Down^TLR2^Up^TLR3^Up^ LTTs) vs. Group B (FFAR2^Up^TLR2^Down^TLR3^Down^ LTTs). Ten gene sets related to cytokines and chemokines were significantly enriched in FFAR2^Down^TLR2^Up^TLR3^Up^ LTTs vs. FFAR2^Up^TLR2^Down^TLR3^Down^ LTTs (**A** and **B**. cytokine receptor activity and binding; **C** and **D**. TNF superfamily cytokine production; **E** and **F**. positive regulation of IL-6 production; **G**. IL-1 pathway; **H** and **I**. IL8-CXCR1 and IL8 CXCR2 pathway; **J**. CXCR4 pathway). NES and the nominal p-value are indicated in each inner panel

FFAR2 deficiency was reported to promote the development of colon adenomas and the progression of adenoma to adenocarcinoma and enhanced the downstream cAMP–PKA–CREB–HDAC pathway [47]. FFAR2 and FFAR3 agonists reduced human monocyte inflammatory cytokine expression by attenuating Akt and ERK2 phosphorylation [48]. Notably, gene sets for cytokine receptor activity and binding (Fig. 3A, B), tumor necrosis factor (TNF) superfamily cytokine production (Fig. 3C, D), positive regulation of IL-6 production (Fig. 3E, F), and the IL-1 pathway (Fig. 3G) were significantly enriched in FFAR2^Down^TLR2^Up^TLR3^Up^ NSCLC (Group A) vs. FFAR2^Up^TLR2^Down^TLR3^Down^ NSCLC (Group B). In addition, gene sets for cytokine-chemokine receptor signaling, such as IL8-CXCR1 (Fig. 3H), IL8-CXCR2 (Fig. 3I), and CXCR4 pathway (Fig. 3J), were also enriched in FFAR2^Down^TLR2^Up^TLR3^Up^ NSCLC (Group A) vs. FFAR2^Up^TLR2^Down^TLR3^Down^ NSCLC (Group B). These results suggest that FFAR2 down-regulation is positively associated with cytokine and chemokine receptor pathways.

### Propionate, an FFAR2 agonist, inhibits TLR2- or TLR3-induced lung cancer progression by inhibiting the AMPK-TAK1 signaling axis for the activation of NF-κB

**Fig. 4:**
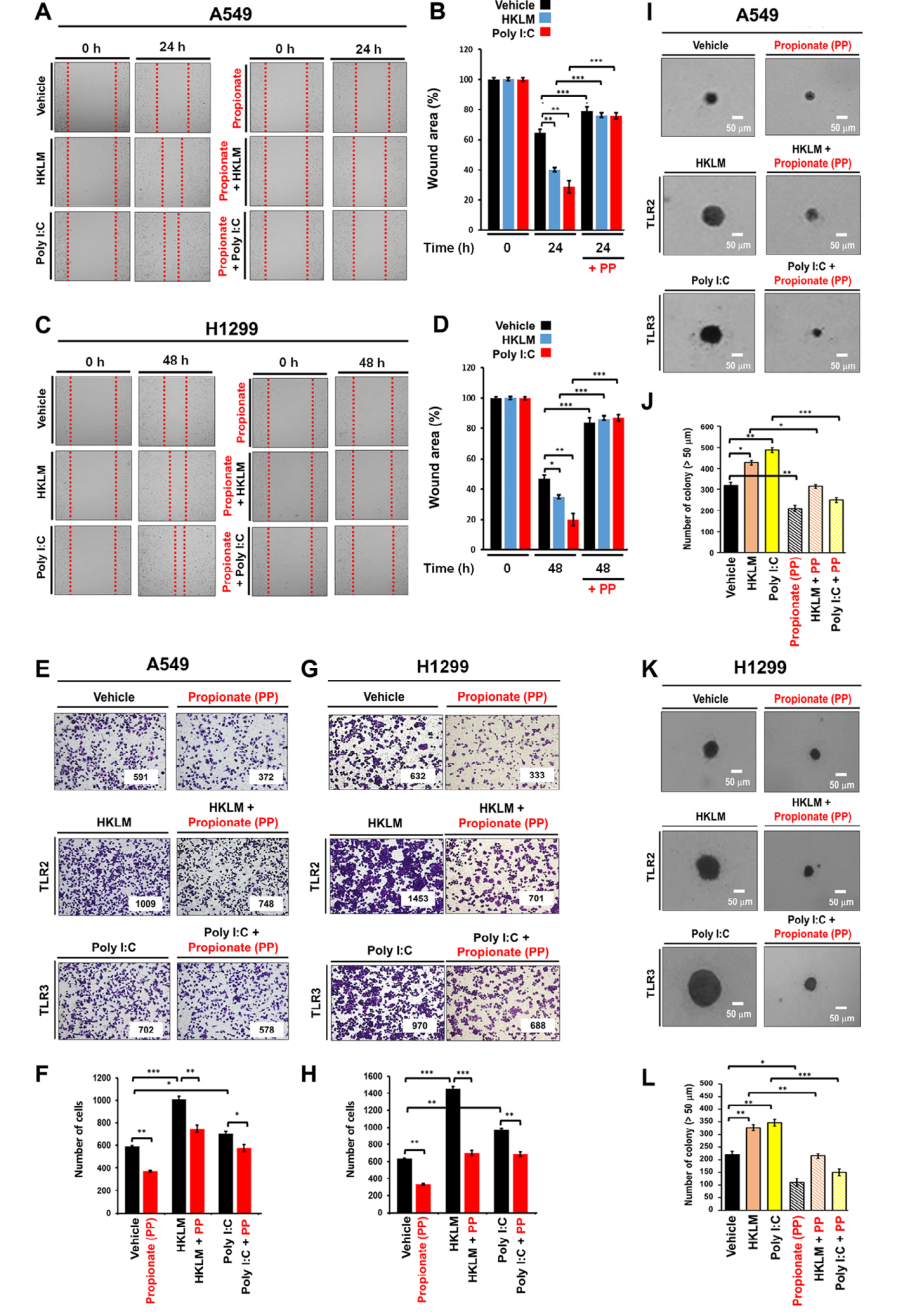
Propionate inhibits TLR2- and TLR3-induced lung cancer progression. **A** and **B**. A549 cells were treated with vehicle, HKLM, and poly(I:C) in the presence or absence of propionate for 24 h, and cell migration was assessed by wound healing assays (**A**). Results are presented as means ± standard deviation (SD, n = 3 independent experiments (**B**). **P < 0.01, ***P < 0.001. **C** and **D**. H1299 cells were treated with vehicle, HKLM, and poly(I:C) in the presence or absence of propionate for 48 h, and cell migration was assessed by wound healing assays (**C**). The results are presented as means ± standard deviation (SD, n = 3 independent experiments) (**D**). *P < 0.05, **P < 0.01, ***P < 0.001. **E** and **F**. A549 cells were treated with vehicle, HKLM, and poly(I:C) in the presence or absence of propionate, as indicated, and cell invasion assays were performed (**E**). The results are presented as means ± standard deviation (SD, n = 3 independent experiments) (**F**). *P < 0.05, **P < 0.01, ***P < 0.001. **G** and **H**. H1299 cells were treated with vehicle, HKLM, and poly(I:C) in the presence or absence of propionate, as indicated, and cell invasion assays were performed (**G**). The results are presented as means ± standard deviation (SD, n = 3 independent experiments (**H**). *P < 0.05, **P < 0.01, ***P < 0.001. **I** and **J**. A549 cells were treated with vehicle, HKLM, and poly(I:C) in the presence or absence of propionate, as indicated, and colony-forming assays were performed (**I**, scale bar: 50 μm). The number of colonies was counted using Adobe Photoshop software, and the results are presented as the mean ± SD of three independent experiments (**J**, n = 3 plates). *P < 0.05, **P < 0.01, and ***P < 0.001. **K** and **L**. H1299 cells were treated with vehicle, HKLM, and poly(I:C) in the presence or absence of propionate, as indicated, and colony-forming assays were performed (**K**, scale bar: 50 μm). The number of colonies was counted using Adobe Photoshop software, and the results are presented as the mean ± SD of three independent experiments (**L**, n = 3 plates). *P < 0.05, **P < 0.01, and ***P < 0.001

Given the above results, we assessed whether FFAR2 functionally inhibited TLR2- or TLR3-induced lung cancer progression. A549 and H1299 lung cancer cells were treated with vehicle, HKLM (an agonist of TLR2), or poly(I:C) (an agonist of TLR3) in the presence or absence of propionate (an agonist of FFAR2), and the cell migration assay was performed. Cell migration was significantly induced by HKLM or poly(I:C) treatment in the absence of propionate (Fig. 4A, B, A549, HKLM or poly(I:C) vs. vehicle; Fig. 4C, D, H1299, HKLM or poly(I:C) vs. vehicle), whereas cell migration was markedly attenuated in the presence of propionate (Fig. 4A, B, A549, HKLM or poly(I:C) vs. HKLM plus propionate or poly(I:C) plus propionate; Fig. 4C, D, H1299, HKLM or poly(I:C) vs. HKLM plus propionate or poly(I:C) plus propionate). Additionally, cell invasion ability was significantly induced by HKLM or poly(I:C) treatment in the absence of propionate (Fig. 4E, F, A549, HKLM or poly(I:C) vs. vehicle; Fig. 4G, H, H1299, HKLM or poly(I:C) vs. vehicle), whereas cell invasion was markedly attenuated in the presence of propionate (Fig. 4E, F, A549, HKLM or poly(I:C) vs. HKLM plus propionate or poly(I:C) plus propionate; Fig. 4G, H, H1299, HKLM or poly(I:C) vs. HKLM plus propionate or poly(I:C) plus propionate). We further performed an in vitro cell survival assay based on the ability of a single cell to grow into a colony. Similar to the cell migration and invasion assay, treatment with HKLM or poly(I:C) induced increases in the size and number of colonies in the absence of propionate (Fig. 4I, J, A549, HKLM or poly(I:C) vs. vehicle; Fig. 4K, L, H1299, HKLM or poly(I:C) vs. vehicle), whereas significant attenuation was observed in the presence of propionate (Fig. 4I, J, A549, HKLM or poly(I:C) vs. HKLM plus propionate or poly(I:C) plus propionate; Fig. 4K, L, H1299, HKLM or poly(I:C) vs. HKLM plus propionate or poly(I:C) plus propionate). These results suggest that the engagement of FFAR2 with propionate antagonizes lung cancer progression induced by TLR2 and TLR3.

**Fig. 5:**
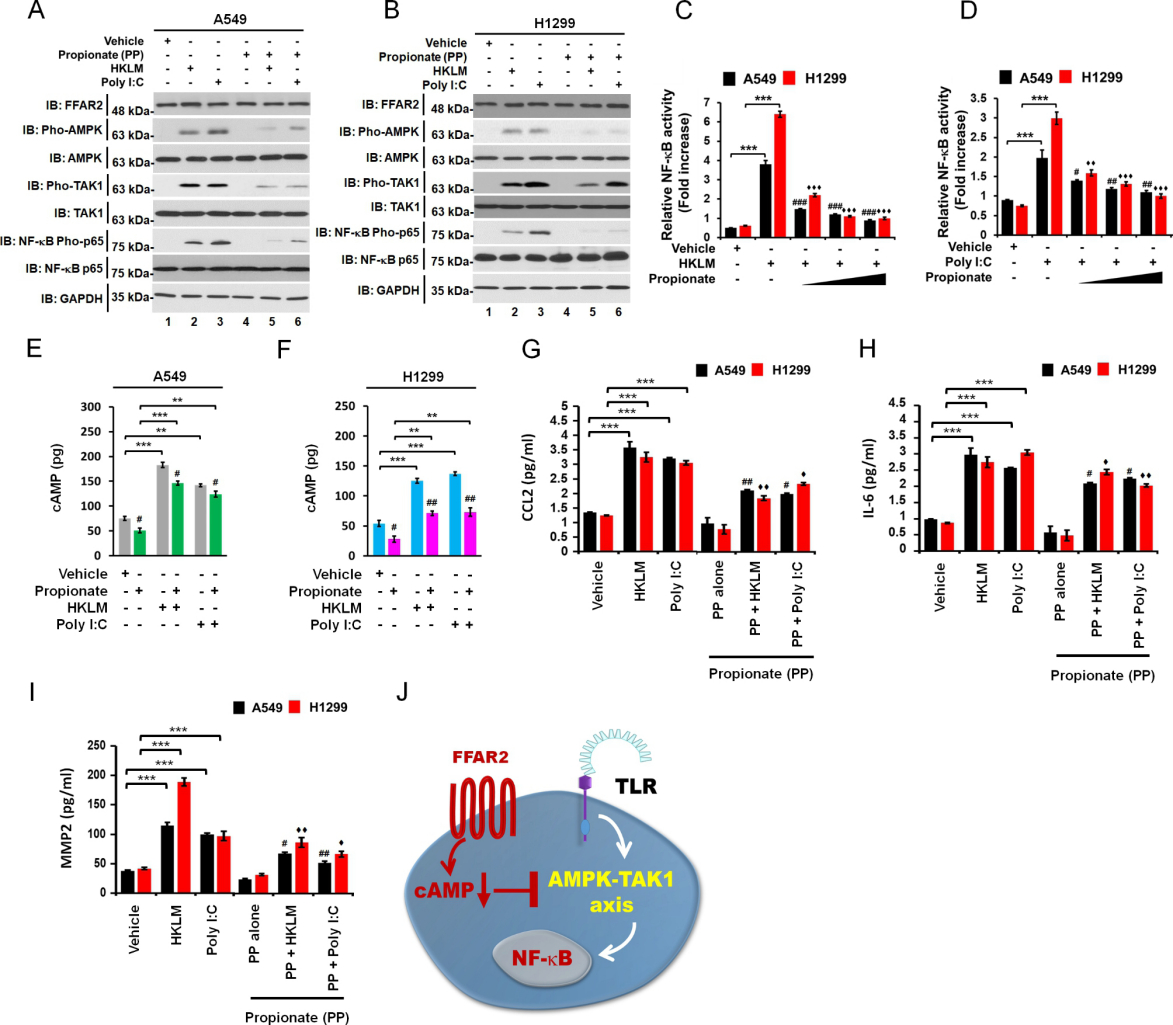
Propionate attenuates the cAMP-AMPK-TAK1 signaling axis for the activation of NF-κB. **A** and **B**. A549 (**A**) and H1299 (**B**) cells were treated with vehicle, HKLM, and poly(I:C) in the presence or absence of propionate, as indicated. The activation of AMPK, TAK1, and p65 was evaluated by pho-AMPK, pho-TAK1, and pho-p65 antibodies. Anti-p65 and anti-GAPDH were used as blot-loading controls. **C** and **D**. A549 and H1299 cells were treated with vehicle, HKLM, and poly(I:C) in the presence or absence of different concentrations of propionate, as indicated. The NF-κB luciferase reporter assay was performed. The results are presented as means ± standard deviation (SD, n = 3 independent experiments). ***P < 0.001; ^#^P < 0.05, ^##^P < 0.01, ^###^P < 0.001, $^{\blacklozenge \blacklozenge}$P < 0.01, and $^{\blacklozenge \blacklozenge \blacklozenge}$P < 0.001 in HKLM or poly(I:C) vs. HKLM plus propionate or poly(I:C) plus propionate. **E** and **F**. A549 (**E**) and H1299 (**F**) cells were treated with vehicle, HKLM, and poly(I:C) in the presence or absence of propionate, as indicated. cAMP levels were measured. The results are presented as means ± standard deviation (SD, n = 3 independent experiments). **P < 0.01 and ***P < 0.001. ^#^P < 0.05 and ^##^P < 0.01 in HKLM or poly(I:C) vs. HKLM or poly(I:C) plus propionate. **G**-**I**. A549 and H1299 cells were treated with vehicle, HKLM, and poly(I:C) in the presence or absence of propionate, as indicated. The production of CCL2 (**G**), IL-6 (**H**), and MMP2 (**I**) cytokines was measured. The results are presented as means ± standard deviation (SD, n = 3 independent experiments). ***P < 0.001; ^#^P < 0.05, ^##^P < 0.01, $^{\blacklozenge }$P < 0.05, and $^{\blacklozenge \blacklozenge}$P < 0.01 in HKLM or poly(I:C) vs. HKLM plus propionate or poly(I:C) plus propionate. **J**. Schematic diagram of how propionate, as a ligand of FFAR2, inhibits the TLR-induced AMPK-TAK1 signaling axis for the activation of NF-κB by modulating cAMP levels

We next explored the molecular mechanism by which FFAR2 signaling inhibited TLR2- and TLR3-induced lung cancer progression. The engagement of TLRs induced NF-κB activation via the TRAF6-TAK1 signaling axis and increases in the production of IL-6, CCL2, CCL20, vascular endothelial growth factor (VEGF), and MMP2, thereby enhancing lung cancer migration and invasion [20]. FFAR2/3 signal induced a decrease in cAMP levels [20]. The cAMP was reported to play a critical role in the activation of NF-κB via PKA signaling and the AMPK-TAK1 signaling axis [49, 50]. Therefore, we hypothesized that FFAR2 may be negatively implicated in the AMPK-TAK1 signaling axis for the activation of NF-κB by the attenuation of cAMP levels and the activation of AMPK. Importantly, co-treatment with propionate significantly attenuated the activation of AMPK, TAK1, and p65 induced by HKLM or poly(I:C) in A549 and H1299 cells compared to those in the absence of propionate (Fig. 5A, A549; Fig. 5B, H1299). Consistently, NF-κB activity was significantly attenuated in A549 and H1299 cells in the presence of different concentrations of propionate (Fig. 5C, HKLM; Fig. 5D, poly(I:C)). Moreover, cAMP levels were increased by HKLM or poly(I:C) treatment, and significantly decreased in the presence of propionate (Fig. 5E, A549; Fig. 5F, H1299). We further assessed whether propionate affected the production of CCL2, IL-6, and MMP2. As expected, the production of these cytokines was enhanced by HKLM or poly(I:C) in A549 and H1299 cells, whereas significant attenuation was observed in the presence of propionate (Fig. 5G, CCL2; Fig. 5H, IL-6; Fig. 5I, MMP2). Taken together, these results suggest that propionate inhibited TLR2- or TLR3-induced lung cancer progression by inhibiting the AMPK-TAK1 signaling axis to activate NF-κB inhibition, as depicted in Fig. 5J.

### FFAR2-knockout (***FFAR2***KO) human lung cancer cells exhibit enhancement of the AMPK-TAK1 signaling axis for the activation of NF-κB

**Fig. 6:**
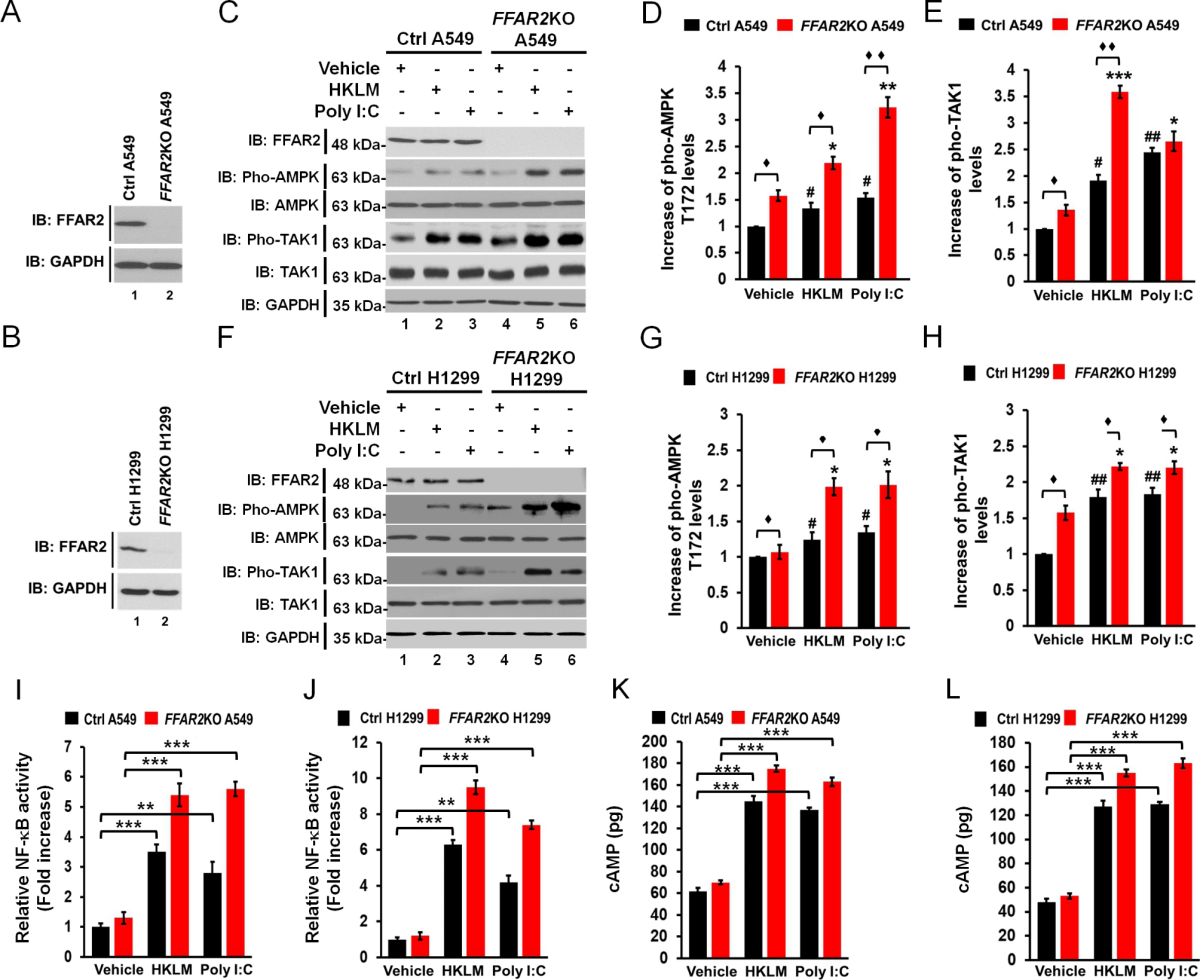
FFAR2-knockout lung cancer cells exhibit enhancement of the cAMP-AMPK-TAK1 signaling axis for the activation of NF-κB in response to TLR2 or TLR3 stimulation. **A** and **B**. *FFAR2*KO A549 (**A**) or *FFAR2*KO H1299 (**B**) cells were generated using the CRISPR/Cas9 method. The expression of FFAR2 was evaluated by Western blotting. **C**-**E**. Ctrl A549 and *FFAR2*KO A549 cells were treated with vehicle, HKLM, and poly(I:C). The activation of AMPK and TAK1 was evaluated by pho-AMPK and pho-TAK1 antibodies (**C**). The band intensity of pho-AMPK (**D**) and pho-TAK1 (**E**) was measured using ImageJ, and normalized with the band intensity of vehicle treatment. The results are presented as means ± standard deviation (SD, n = 3 independent experiments). $^{\blacklozenge }$P < 0.05 and $^{\blacklozenge \blacklozenge}$P < 0.01. (**F**-**H)**. Ctrl H1299 and *FFAR2*KO H1299 cells were treated with vehicle, HKLM, and poly(I:C). The activation of AMPK and TAK1 was evaluated by pho-AMPK and pho-TAK1 antibodies (**F**). The bend intensity of pho-AMPK (**G**) and pho-TAK1 (**H**) was measured using ImageJ. The results are presented as means ± standard deviation (SD, n = 3 independent experiments). $^{\blacklozenge }$P < 0.05. **I** and **J**. Ctrl A549 and *FFAR2*KO A549 (**I**) or Ctrl H1299 and *FFAR2*KO H1299 (**J**) cells were treated with vehicle, HKLM, and poly(I:C), as indicated, and the NF-κB luciferase reporter assay was performed. The results are presented as means ± standard deviation (SD, n = 3 independent experiments). **P < 0.01, and ***P < 0.001. **K** and **L**. Ctrl A549 and *FFAR2*KO A549 (**K**) or Ctrl H1299 and *FFAR2*KO H1299 (**L**) cells were treated with vehicle, HKLM, and poly(I:C), as indicated, and cAMP levels were measured. The results are presented as means ± standard deviation (SD, n = 3 independent experiments). ***P < 0.001

Given the above results, we tried to verify the functional role of the FFAR2 on the AMPK-TAK1 signaling axis in the activation of NF-κB. We generated *FFAR2*-knockout A549 and H1299 cells using the CRISPR/cas9 gene-editing method (Supplementary Figures S4A and S4B; Fig. 6A, *FFAR2*KO A549; Fig. 6B, *FFAR2*KO H1299). Treatment with HKLM or poly(I:C) induced the activation of AMPK and TAK1 in control (Ctrl) A549 and Ctrl H1299 cells (Fig. 6C-E, Ctrl A549; Fig. 6F-H, Ctrl H1299). Importantly, the pho-levels of AMPK and TAK1 were markedly enhanced in *FFAR2*KO A549 and *FFAR2*KO H1299 cells compared to those in Ctrl A549 and Ctrl H1299 cells (Fig. 6C-E, *FFAR2*KO A549 vs. Ctrl A549; Fig. 6F-H, *FFAR2*KO H1299 vs. Ctrl H1299). NF-κB activity was increased in Ctrl A549 and Ctrl H1299 cells by HKLM or poly(I:C) treatment (Fig. 6I, HKLM or poly(I:C) vs. vehicle in Ctrl A549; Fig. 6J, HKLM or poly(I:C) vs. vehicle in Ctrl H1299), and markedly enhanced in *FFAR2*KO A549 and *FFAR2*KO H1299 cells (Fig. 6I, *FFAR2*KO A549 vs. Ctrl A549; Fig. 6J, *FFAR2*KO H1299 vs. Ctrl H1299). Additionally, the levels of cAMP were increased in Ctrl A549 and Ctrl H1299 cells by HKLM or poly(I:C) (Fig. 6K, HKLM or poly(I:C) vs. vehicle in Ctrl A549; Fig. 6L, HKLM or poly(I:C) vs. vehicle in Ctrl H1299), and significantly elevated in *FFAR2*KO A549 and *FFAR2*KO H1299 cells (Fig. 6K, *FFAR2*KO A549 vs. Ctrl A549; Fig. 6L, *FFAR2*KO H1299 vs. Ctrl H1299). These results suggest that the deficiency of FFAR2 enhances NF-κB activation by TLR2- or TLR3- via the activation of the AMPK-TAK1 signaling axis.

### ***FFAR2***KO lung cancer cells enhance cancer progression induced by TLR2 and TLR3

**Fig. 7:**
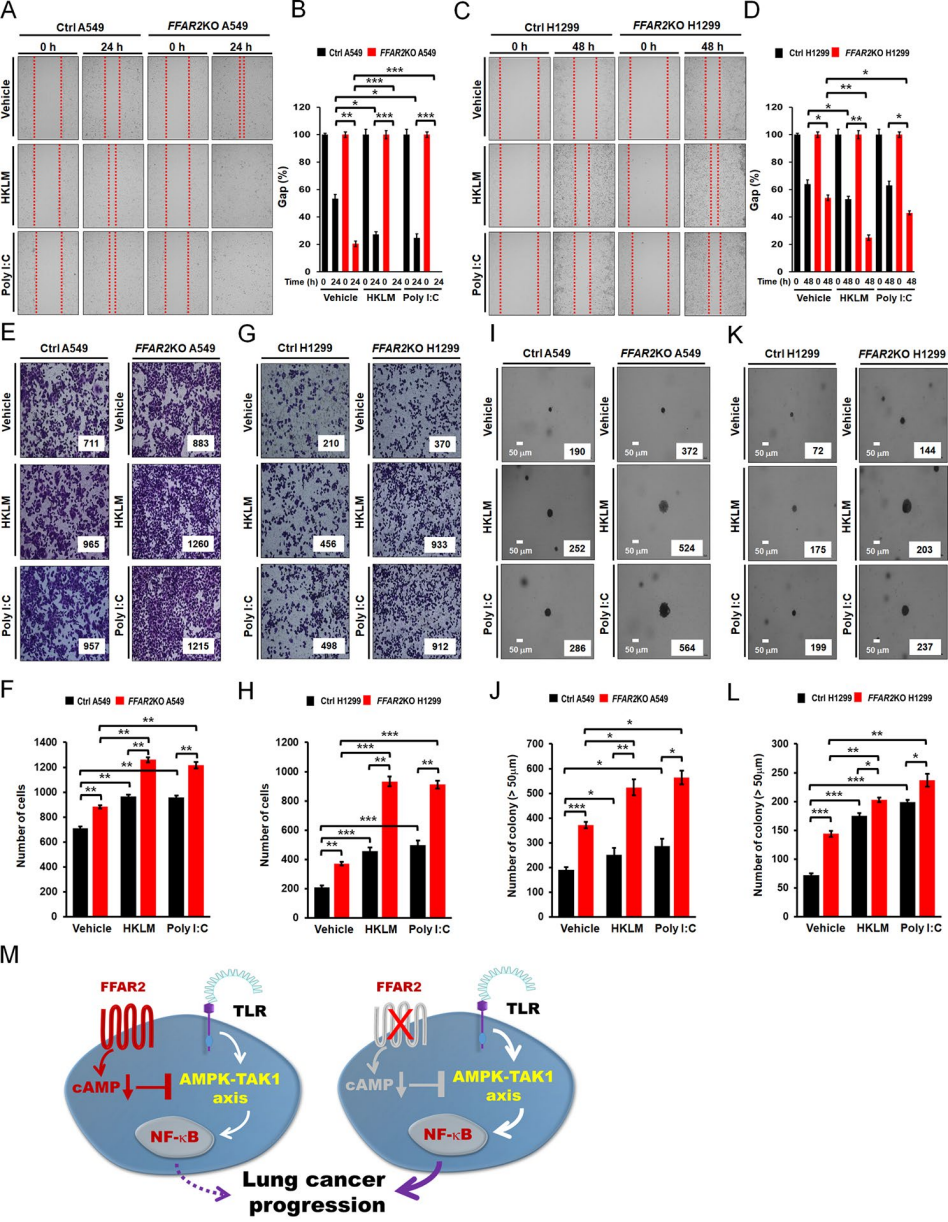
*FFAR2*KO lung cancer cells exhibit the enhancement of cancer migration, invasion, and colony formation induced by TLR2 or TLR3. **A** and **B**. Ctrl A549 and *FFAR2*KO A549 cells were treated with vehicle, HKLM, and poly(I:C) for 24 h. Cell migration was assessed by the wound healing assay (**A**). The results are presented as means ± standard deviation (SD, n = 3 independent experiments (**B**). *P < 0.05, **P < 0.01, and ***P < 0.001. **C** and **D**. Ctrl H1299 and *FFAR2*KO H1299 cells were treated with vehicle, HKLM, and poly(I:C) for 48 h. Cell migration was assessed by the wound healing assay (**C**). The results are presented as means ± standard deviation (SD, n = 3 independent experiments (**D**). *P < 0.05 and **P < 0.01. **E** and **F**. Ctrl A549 and *FFAR2*KO A549 cells were treated with vehicle, HKLM, and poly(I:C), and the cell invasion assay was performed (**E**). The results are presented as means ± standard deviation (SD, n = 3 independent experiments) (**F**). **P < 0.01 and ***P < 0.001. **G** and **H**. Ctrl H1299 and *FFAR2*KO H1299 cells were treated with vehicle, HKLM, and poly(I:C), and the cell invasion assay was performed (**G**). The results are presented as means ± standard deviation (SD, n = 3 independent experiments) (**H**). **P < 0.01 and ***P < 0.001. **I** and **J**. Ctrl A549 and *FFAR2*KO A549 cells were treated with vehicle, HKLM, and poly(I:C), and the colony-forming assay was performed (**I**, scale bar: 50 μm). The number of colonies was counted using Adobe Photoshop software, and the results are presented as the mean ± SD of three independent experiments (**J**, n = 3 plates). *P < 0.05, **P < 0.01, and ***P < 0.001. **K** and **L**. Ctrl H1299 and *FFAR2*KO H1299 cells were treated with vehicle, HKLM, and poly(I:C), and the colony-forming assay was performed (**K**, scale bar: 50 μm). The number of colonies was counted using Adobe Photoshop software, and the results are presented as the mean ± SD of three independent experiments (**L**, n = 3 plates). *P < 0.05, *P < 0.01, and ***P < 0.001. **M**. Schematic model of regulation by FFAR2 in TLR-induced lung cancer progression through the cAMP-AMPK-TAK1 signaling axis for NF-κB activation

We finally examined whether FFAR2 deficiency enhanced lung cancer progression. Ctrl A549, *FFAR2*KO A549, Ctrl H1299, and *FFAR2*KO H1299 cells were treated with vehicle, HKLM, and poly(I:C). Cancer progression ability was assessed by cancer migration, invasion, and colony-forming assays. As expected, treatment with HKLM or poly(I:C) induced increases in cell migration compared to vehicle treatment (Fig. 7A, B, HKLM or poly(I:C) vs. vehicle in Ctrl A549; Fig. 7C, D, HKLM or poly(I:C) vs. vehicle in Ctrl H1299). Importantly, cell migration was significantly enhanced in *FFAR2*KO A549 and *FFAR2*KO H1299 cells compared to Ctrl A549 and Ctrl H1299 cells, respectively (Fig. 7A, B, *FFAR2*KO A549 treated with HKLM or poly(I:C) vs. Ctrl A549 treated with HKLM or poly(I:C); Fig. 7C, D, *FFAR2*KO H1299 treated with HKLM or poly(I:C) vs. Ctrl H1299 treated with HKLM or poly(I:C)). Consistently, cell invasion ability was also elevated in *FFAR2*KO A549 and *FFAR2*KO H1299 cells compared to Ctrl A549 and Ctrl H1299 cells, respectively (Fig. 7E, F, *FFAR2*KO A549 treated with HKLM or poly(I:C) vs. Ctrl A549 treated with HKLM or poly(I:C); Fig. 7G, H, *FFAR2*KO H1299 treated with HKLM or poly(I:C) vs. Ctrl H1299 treated with HKLM or poly(I:C)). In addition, the production of CCL2, IL-6, and MMP2 cytokines was significantly enhanced in *FFAR2*KO A549 and *FFAR2*KO H1299 cells treated with HKLM or poly(I:C) compared to Ctrl A549 and Ctrl H1299 cells (Supplementary Figures S5A-S5C, *FFAR2*KO A549 vs. Ctrl A549; Supplementary Figures S5D-S5F, *FFAR2*KO H1299 vs. Ctrl H1299). Moreover, the colony-forming assay revealed increases in the number of colonies in *FFAR2*KO A549 and *FFAR2*KO H1299 cells treated with HKLM or poly(I:C) compared to Ctrl A549 and Ctrl H1299 cells (Fig. 7I, J, *FFAR2*KO A549 treated with HKLM or poly(I:C) vs. Ctrl A549 treated with HKLM or poly(I:C); Fig. 7K, L, *FFAR2*KO H1299 treated with HKLM or poly(I:C) vs. Ctrl H1299 treated with HKLM or poly(I:C)). Taken together, these results suggest that FFAR2 antagonizes TLR2- and TLR3-induced lung cancer progression.

## Discussion

In this study, we demonstrated that FFAR2 was negatively involved in lung cancer progression induced by TLR2 and TLR3 through the suppression of the cAMP-AMPK-TAK1 signaling axis for the activation of NF-κB. Clinical TCGA data and our cohort datasets (NSCLCs, n = 42) revealed a reverse correlation between FFAR2 and TLR2 or TLR3 expression. Moreover, GSEA showed significant enrichments in gene sets related to the cancer module, the innate signaling pathway, and the cytokine-chemokine signaling pathway in FFAR2^Down^TLR2^Up^TLR3^Up^ NSCLC LTTs vs. FFAR2^up^TLR2^Down^TLR3^Down^ NSCLC LTTs. Functionally, treatment with propionate (an agonist of FFAR2) significantly inhibited human lung cancer migration, invasion, and colony formation induced by TLR2 or TLR3 by attenuating the cAMP-AMPK-TAK1 signaling axis for the activation of NF-κB. Notably, *FFAR2*KO human lung cancer *FFAR2*KO A549 and *FFAR2*KO H1299 cells showed marked increases in cell migration, invasion, and colony formation in response to TLR2 or TLR3 stimulation, accompanied by elevations in NF-κB activation, cAMP levels, and the production of CCL2, IL-6, and MMP2 cytokines. Collectively, our results strongly suggest the FFAR2 signal might antagonize TLR2- and TLR3-induced lung cancer progression via suppression of the cAMP-AMPK-TAK1 signaling axis for the activation of NF-κB.

Recently, the relationship between TLRs and lung cancer progression has become an important issue in terms of pathophysiology and the development of therapeutic targets [22, 23]. Since chronic and persistent inflammation is associated with a higher risk of cancer development, many studies have focused on TLR expression in lung cancer [22, 51, 52]. The expression of TLR4, 5, 7, 8, and 9 in NSCLC was reported to be markedly higher than in normal lung tissue [22, 27–29]. In addition, the activation of TLR2, TLR3, and TLR4 was functionally implicated in lung cancer proliferation, migration, and invasion [20, 53]. In terms of biochemical mechanisms, NF-κB activation by the engagement of TLRs plays a pivotal role in cancer proliferation, survival, angiogenesis, and progression through the up-regulation of IL-6, Bcl-xL, Bcl-2, Bcl-xs, XIAP, and VEGF genes [20, 23, 54]. Therefore, the signaling pathway of TLR-induced NF-κB activation is being considered a potential target for lung cancer treatment intervention. We attempted to analyze the association between FFARs and TLRs in the TCGA lung cancer dataset and our NSCLC patient dataset (n = 42). We found that, of the FFARs, FFAR2 was negatively correlated with TLR2 and TLR3. Moreover, GSEA between FFAR2^Down^TLR2^Up^TLR3^Up^ LTTs and FFAR2^Up^TLR2^Down^TLR3^Down^ LTTs of NSCLC patients revealed significant enrichments in the cancer module, innate signaling pathways, and cytokine-chemokines gene sets in FFAR2^Down^TLR2^Up^TLR3^Up^ LTTs, suggesting a functional association between FFAR2 and TLR2 or TLR3 in lung cancer.

Accumulating evidence has shown that SCFAs as ligands of FFARs can influence the development and progression of various cancers, including colorectal, bladder, breast, gastric, liver, and lung cancer [7–9]. In lung cancer, reduced numbers of microbiome organisms, such as *Enterobacter, Dialister, Fecalibacterium, Kluyvera, Escherichia–Shigella, Fusobacterium, Bacteroides*, and *Veillonella*, were found in lung cancer patients compared to controls [55]. SCFAs can trigger cascades of responses that lead either to malignancy or hinder cancer through the stimulation of GPCRs [56]. Most studies on SCFAs have focused on butyrate, while the function of propionate in lung cancer is not well established [57]. A previous study showed that treatment with propionate exhibited anticancer properties for lung cancer by activating cell apoptosis and cell cycle arrest by reducing survivin expression and increasing p21 expression [57]. We found that propionate inhibited TLR2- and TLR3-induced lung cancer migration, invasion, and colony formation accompanied by the inhibition of the cAMP-AMPK-TAK1 signaling axis for the activation of NF-κB and the production of CCL2, IL-6, and MMP2 cytokines. The engagement of TLR3/4 was reported to induce increases in the production of IL-6, CCL2, CCL20, VEGF, and MMP2 through NF-κB activation, thereby enhancing lung cancer migration and invasion [20]. Additionally, SCFAs acting through FFAR2 inhibited cAMP production by protein kinase A (PKA) activity and the expression of IL-6, IL-1β, and TNFα to exert anti-inflammatory effects [58, 59]. Importantly, we found that *FFAR2*KO A549 and *FFAR2*KO H1299 lung cancer cells exhibited the enhancement of cell migration, invasion, and colony formation induced by TLR2 and TLR3, along with increases in the production of CCL2, IL-6, and MMP2 cytokines. Biochemical studies revealed significant elevations in cAMP levels and the activation of AMPK and TAK1 for the activation of NF-κB in response to TLR2 and TLR3. Considering these previous results [20, 58, 59], our results suggest that propionate acting through FFAR2 might antagonize the cAMP-AMPK-TAK1 signaling axis induced by TLR2 and TLR3 for the activation of NF-κB.

## Conclusion

In summary, our study identified negative regulation by propionate, which is an SCFA and recognized by FFAR2, in TLR2- and TLR3-induced lung cancer progression. As depicted in Fig. 7M, the engagement of propionate with FFAR2 induced decreases in cAMP levels, resulting in the attenuation of the AMPK-TAK1 signaling axis for the activation of NF-κB (Fig. 7M, left). Since it has been reported that cAMP-dependent protein kinase A (PKA) mediated the phosphorylation of TAK1 at Ser412 for the activation of NF-κB [60], the decrease in cAMP by propionate might have a negative effect on TLR2- and TLR3-induced activation of TAK1 for NF-κB activation (Fig. 7M, left). In contrast, the down-regulation of FFAR2 in lung cancer did not attenuate the cAMP-AMPK-TAK1 signaling axis for the activation of NF-κB, thereby enhancing TLR2- and TLR3-induced lung cancer progression (Fig. 7M, right). Taken together, our results might contribute to a better understanding of the pathophysiology and progression of lung cancer regulated by microbiome-derived metabolites and PAMPs, and thereby, the development of therapeutics for lung cancer.

## Electronic supplementary material

Below is the link to the electronic supplementary material.

Supplementary Material 1

## Data Availability

All data that support the findings of this study are available from the corresponding authors upon reasonable request.

## Abbreviations

FFARs: Free fatty acid receptors
TLRs: Toll-like receptors
TCGA: The cancer genome atlas
NSCLC: Non-small cell lung cancer
GSEA: Gene set enrichment analysis
LTT: Lung tumor tissue
LNT: Lung normal tissue
cAMP: Cyclic adenosine monophosphate
AMPK: AMP-activated protein kinase
TAK1: Transforming growth factor-β-activated kinase 1
NF-κB: Nuclear factor-κB
IL-6: Interleukin 6
CCL2: C-C motif chemokine ligand 2
VEGF: Vascular endothelial growth factor
MMP2: Matrix metalloproteinase 2
SCFAs: Short-chain fatty acids
PAMPs: Pathogen-associated molecular patterns
TME: Tumor microenvironment
IBD: Inflammatory bowel disease
PRRs: Pattern recognition receptors
NLRs: Nucleotide oligomerization domain (NOD)-like receptors
RLRs: Retinoic acid-inducible gene (RIG-1)-like receptors
