# Supplementary material for: FFAR2 antagonizes TLR2- and TLR3-induced lung cancer progression via the inhibition of AMPK-TAK1 signaling axis for the activation of NF-κB
Source: Cell Biosci. 2023 Jun 7;13:102. doi: 10.1186/s13578-023-01038-y (PMC10249240; doi:10.1186/s13578-023-01038-y)
Supplement: Supplementary file 1 — Supplementary Material 1 [file 13578_2023_1038_MOESM1_ESM.docx]

**Supplementary Information**

**FFAR2 antagonizes TLR2- and TLR3-induced lung cancer progression via the inhibition of AMPK-TAK1 signaling axis for the activation of NF-κB**

**Methods**

**Gene Expression Profiling Interactive Analysis**

FFAR1, FFAR3, and FFAR4 expression in LUAD and LUSC was analyzed by using gene expression profiling interactive analysis (GEPIA, <http://gepia.cancer-pku.cn/detail.php?gene=FFAR1>, <http://gepia.cancer-pku.cn/detail.php?gene=FFAR3>, <http://gepia.cancer-pku.cn/detail.php?gene=FFAR4>) data. Correlation analysis between FFAR2 expression and TLR1, TLR4, TLR6, TLR7, TLR8, or TLR9 was performed in lung adenocarcinoma (LUAD) by using GEPIA ( <http://gepia.cancer-pku.cn/detail.php?gene=FFAR2>).

**Measurement of CCL2, IL-6, and MMP2 cytokines**

The production of CCL2, IL-6, and MMP2 cytokines was measured as previously described [1]. Briefly, Ctrl A549, *FFAR2*KO A549, Ctrl H1299, and *FFAR2*KO H1299 cells were treated with vehicle (DMSO, 0.1% v/v concentration), HKLM (10^8^ cells/mL), and poly(I:C) (20 μg/mL) for 24 h. CCL2 (DCP00), IL-6 (D6050), and MMP2 (DMP2F0) levels in the supernatant fractions were measured by ELISA (R&D Systems) according to the manufacturer’s protocols.

**References**

1. Zhan Z, Xie X, Cao H, Zhou X, Zhang XD, Fan H, et al. Autophagy facilitates TLR4- and TLR3-triggered migration and invasion of lung cancer cells through the promotion of TRAF6 ubiquitination. Autophagy. 2014;10(2):257-68

**Supplementary Figure S1.**


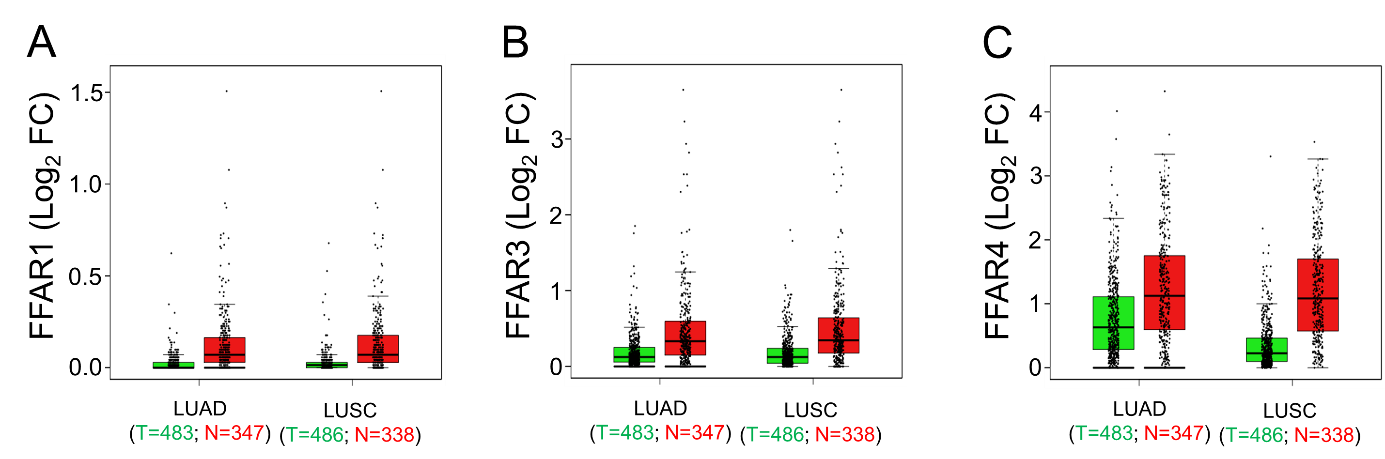


**Fig. S1** FFAR1, FFAR3, and FFAR4 expression in lung cancer.

**A**-**C**. FFAR1 (**A**), FFAR3 (**B**), and FFAR4 (**C**) expression was compared between tumor (T) and normal (N) tissues by using gene expression profiling interactive analysis (GEPIA, <http://gepia.cancer-pku.cn/detail.php?gene=FFAR1>, <http://gepia.cancer-pku.cn/detail.php?gene=FFAR3>, <http://gepia.cancer-pku.cn/detail.php?gene=FFAR4>) data.

**Supplementary Figure S2.**


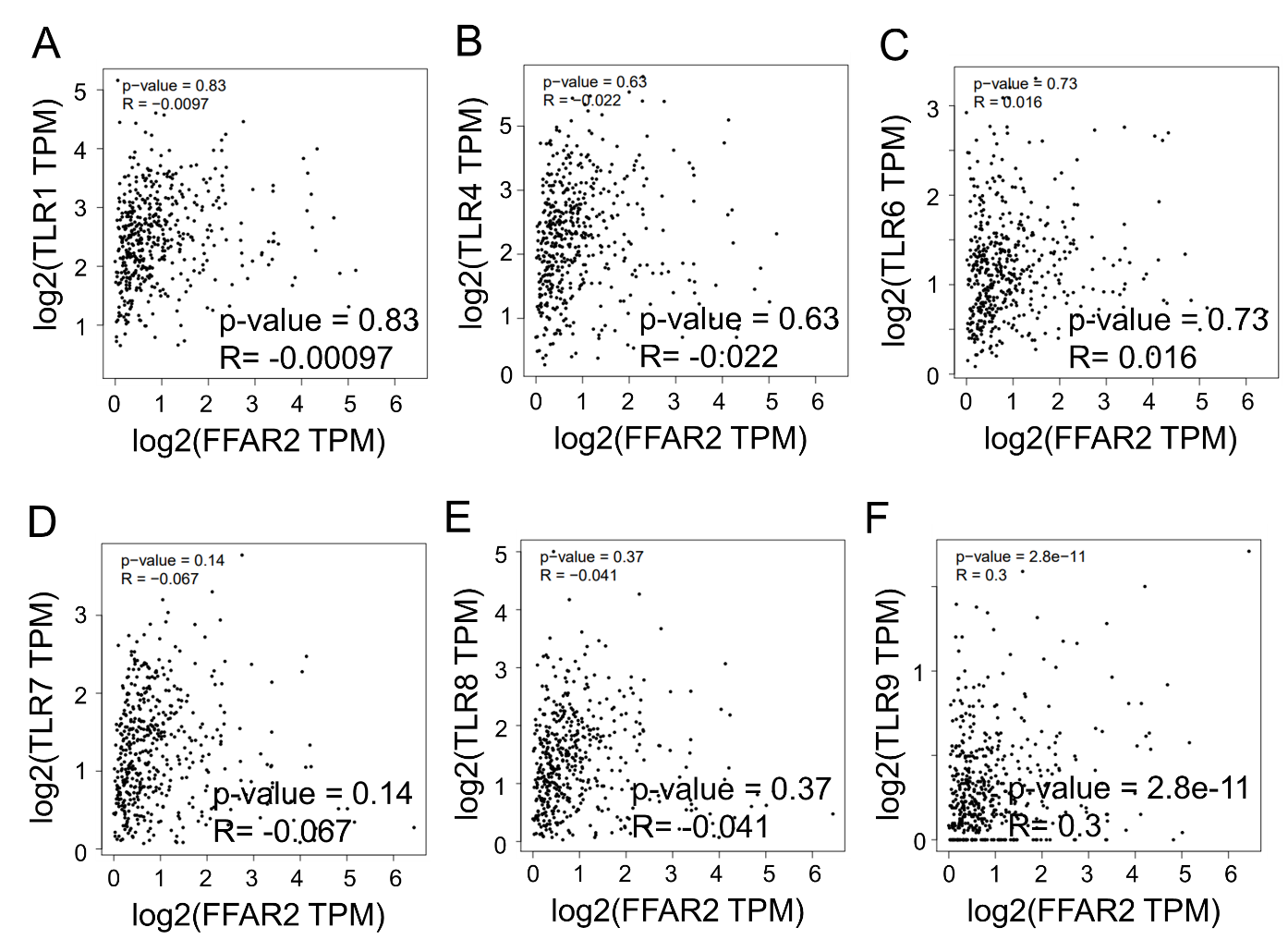


**Fig. S2** Correlation analysis between FFAR2 expression and TLR1, TLR4, TLR6, TLR7, TLR8, or TLR9. **A-F**. Correlation analysis between FFAR2 expression and TLR1 (**A**), TLR4 (**B**), TLR6 (**C**), TLR7 (**D),** TLR8 (**E**), or TLR9 (**F**) in lung adenocarcinoma (LUAD) was performed by using gene expression profiling interactive analysis (GEPIA, <http://gepia.cancer-pku.cn/detail.php?gene=FFAR2>). R value was inserted in each panel.

**Supplementary Figure S3.**


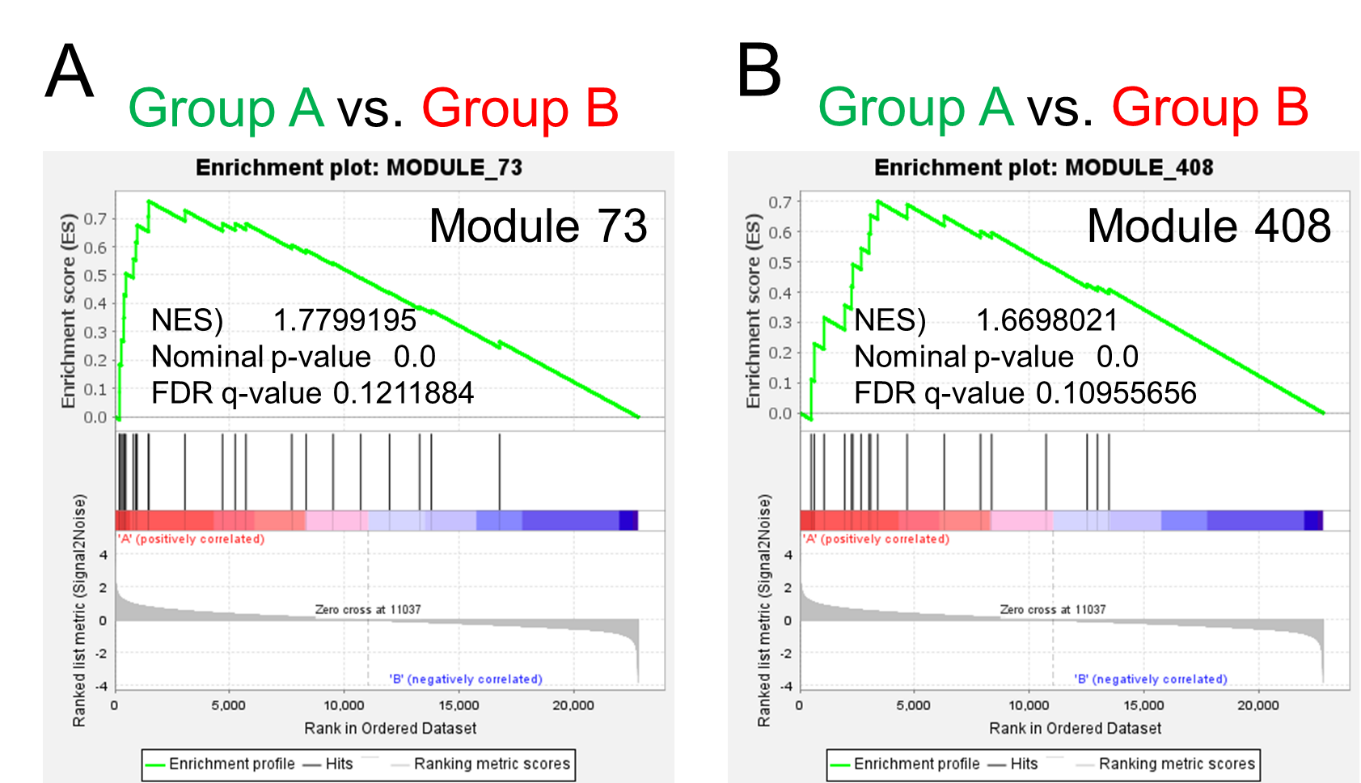


**Fig. S3** GSEA was performed in FFAR2^Down^TLR2^Up^TLR3^Up^ LTTs vs. FFAR2^Up^TLR2^Down^TLR3^Down^ LTTs. **A** and **B**. GSEA (<http://www.gsea-msigdb.org/gsea/index.jsp>) was performed for Group A (FFAR2^Down^TLR2^Up^TLR3^Up^ LTTs) vs. Group B (FFAR2^Up^TLR2^Down^TLR3^Down^ LTTs). Three gene sets for cancer modules were significantly enriched in Group A vs. Group B (**A**, module 73 and **B**, module 408). NES and nominal p-value are indicated in each inner panel.

**Supplementary Figure S4.**


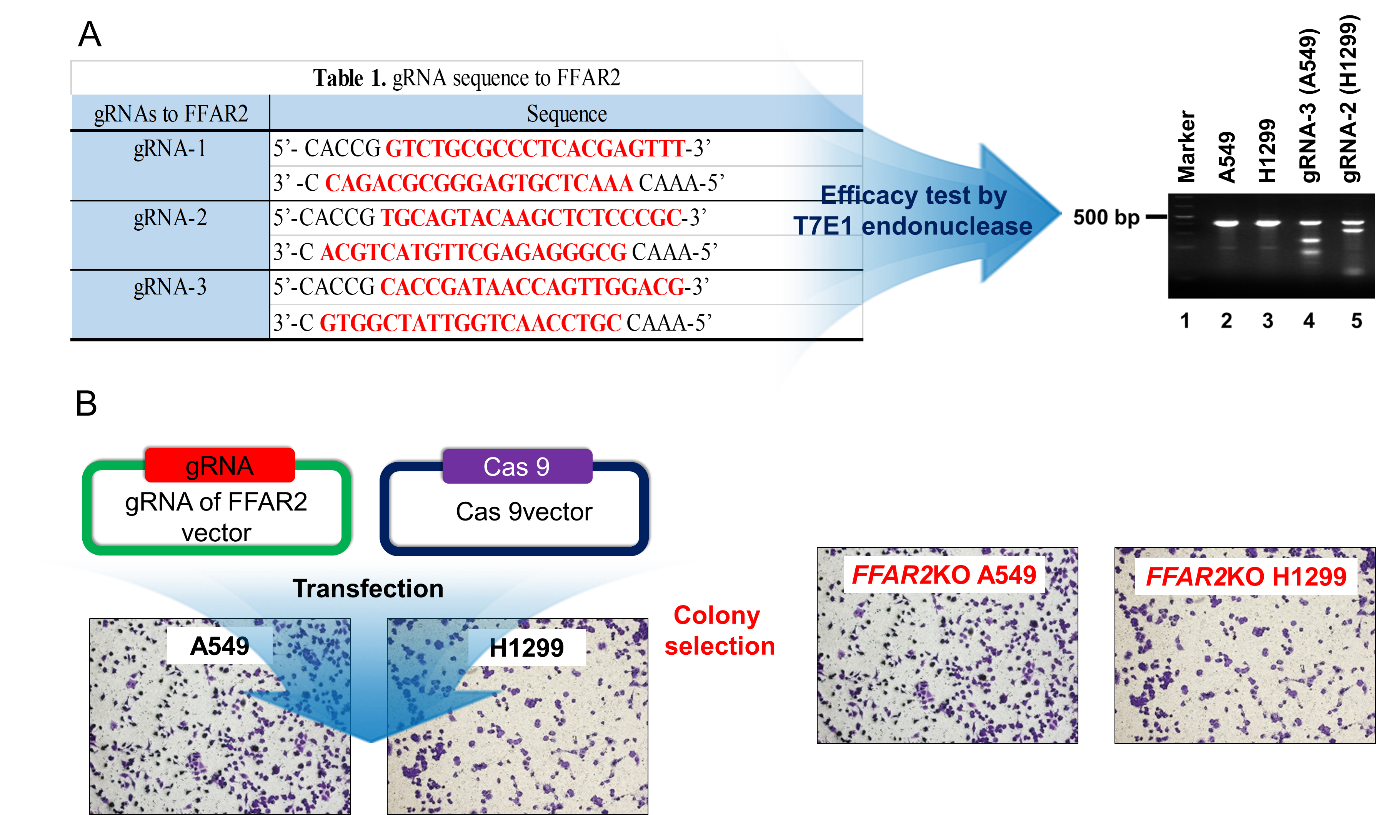


**Fig. S4** Schematic view of the generation of FFAR2-knockout lung cancer cells by using CRISPR/Cas9 method. **A.** FFAR2-guide RNA sequences for CRISPR/Cas9 were designed. Guide RNA sequences for *FFAR2* were gRNA1, 5’-CACCGGTCTGCGCCCTCACGAGTTT-3’ and 3’-CCAGACGCGGGAGTGCTCAAACAAA-5’; gRNA2, 5’-CACCG TGCAGTACAAGCTCTCCCGC-3’ and 3’-CACGTCATGTTCGAGAGGGCGCAAA-5’; gRNA3, 5’-CACCGCACCGATAACCAGTTGGACG-3’ and 3’-CGTGGCTATTGGTCAACCTGCCAAA-5’. And the efficacy of gRNA-3 and gRNA-2 was tested in A549 and H1299 cells, respectively, by T7E1 endonuclease. **B**. By using two vector system, sgRNA and cas9 vectors, *FFAR2*KO A549 and *FFAR2*KO H1299 cells were generated.

**Supplementary Figure S5.**


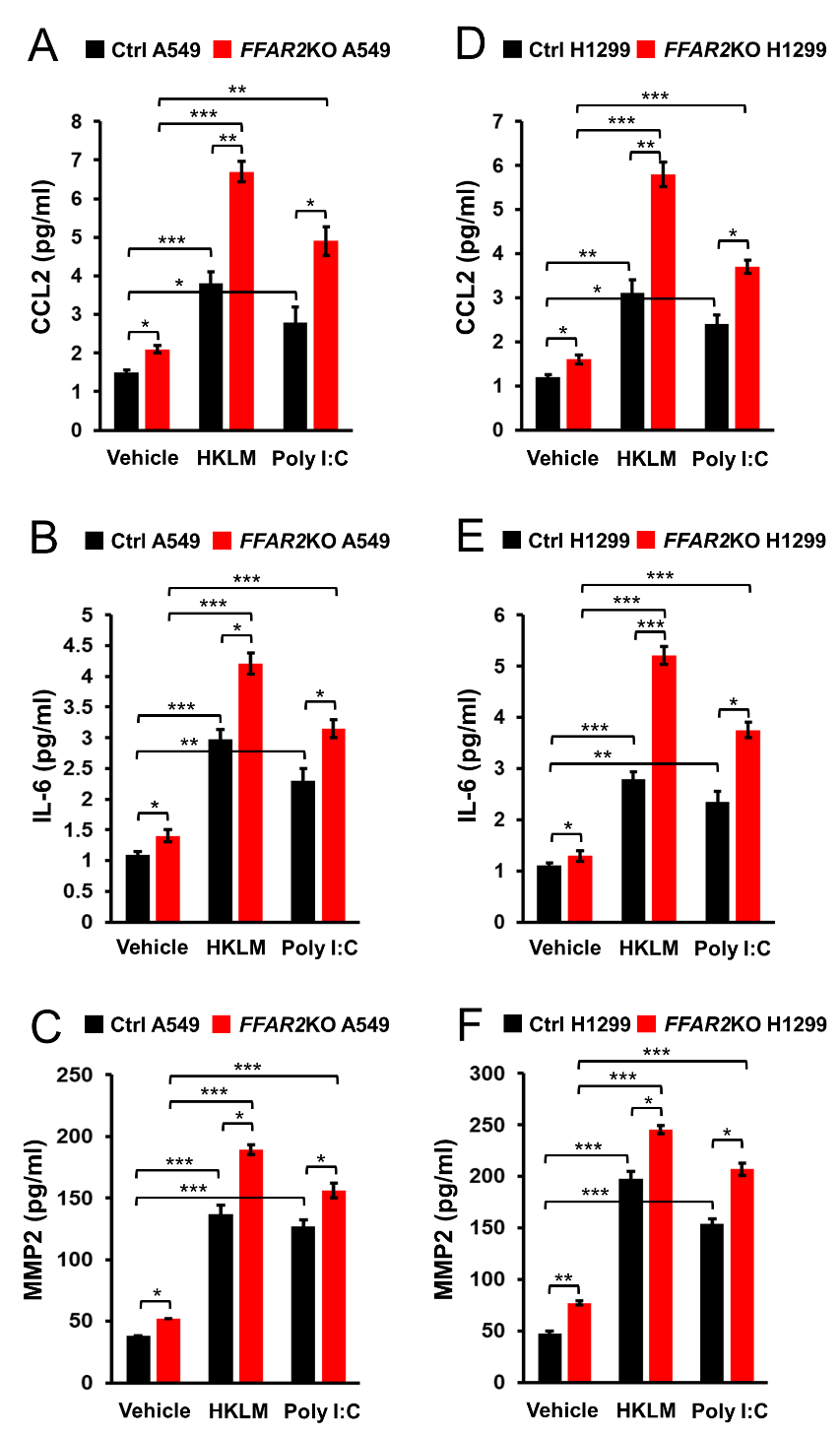


**Fig. S5** Production of CCL2, IL-6, and MMP2 was elevated in *FFAR2*KO A549 or *FFAR2*KO H1299 cells in response to TLR2 and TLR3. **A**-**C**. Ctrl A549 and *FFAR2*KO A549 cells were treated with vehicle, HKLM, and Poly I:C, as indicated. Production of CCL2 (**A**), IL-6 (**B**), and MMP2 (**C**) cytokines was measured. Results were represented as means ± standard deviation (SD, n=3 independent experiments). * *P*< 0.05, ** *P*< 0.01, and *** *P*< 0.001. **D**-**F**. Ctrl H1299 and *FFAR2*KO H1299 cells were treated with vehicle, HKLM, and Poly I:C, as indicated. Production of CCL2 (**D**), IL-6 (**E**), and MMP2 (**F**) cytokines was measured. Results were represented as means ± standard deviation (SD, n=3 independent experiments). * *P*< 0.05, ** *P*< 0.01, and *** *P*< 0.001.

| **Supplementary Table S1. Magnitude difference between lung tumor tissues (LTTs) and matched lung normal tissues (mLNTs)** | | | | | | | | | | |
| --- | --- | --- | --- | --- | --- | --- | --- | --- | --- | --- |
| lung tumor tissues (LTTs) | | | | matched lung normal tisseus (mLNTs) | | | | **∆Mag = LTT Mag – mLNT Mag** | | |
| LTTs | FFAR2 | TLR2 | TLR3 | mLNTs | FFAR2 | TLR2 | TLR3 | **∆FFAR2** | **∆TLR2** | **∆TLR3** |
| LTT20.AVG_Signal | 60.47514 | 24.00914 | 11.49744 | LNT20.AVG_Signal | -15.119 | -3.25836 | 7.504193 | **75.59418** | **27.267501** | **3.993247** |
| LTT07.AVG_Signal | 48.04034 | -21.2879 | 33.83842 | LNT07.AVG_Signal | -12.0953 | -9.69048 | 29.12931 | **60.13559** | **-11.59743** | **4.70911** |
| LTT26.AVG_Signal | 31.99884 | -11.7817 | 1.710847 | LNT26.AVG_Signal | -11.6919 | 16.03239 | 23.74983 | **43.69078** | **-27.81404** | **-22.038983** |
| LTT01.AVG_Signal | 51.52975 | 6.050843 | 54.54247 | LNT01.AVG_Signal | 8.420033 | -9.694 | 12.60587 | **43.109717** | **15.744846** | **41.9366** |
| LTT06.AVG_Signal | 22.31039 | -10.5663 | 10.25804 | LNT06.AVG_Signal | -5.00048 | -10.8532 | 9.197865 | **27.310866** | **0.28691** | **1.060175** |
| LTT42.AVG_Signal | 10.89388 | 8.191467 | 25.93987 | LNT42.AVG_Signal | -6.42182 | 2.912867 | 30.1293 | **17.315696** | **5.2786** | **-4.18943** |
| LTT51.AVG_Signal | 14.98341 | 55.19246 | 48.83957 | LNT51.AVG_Signal | -1.94687 | 6.901487 | 44.86845 | **16.930277** | **48.290973** | **3.97112** |
| LTT04.AVG_Signal | 11.17395 | -22.2535 | 21.70125 | LNT04.AVG_Signal | -3.36537 | 0.934711 | 31.64551 | **14.539319** | **-23.1882205** | **-9.94426** |
| LTT02.AVG_Signal | 14.4069 | -14.5028 | 36.52794 | LNT02.AVG_Signal | 1.895533 | -4.13712 | 31.6799 | **12.511367** | **-10.365639** | **4.84804** |
| LTT24.AVG_Signal | 8.646358 | 24.0082 | 39.82794 | LNT24.AVG_Signal | -3.01593 | 6.157336 | 65.35287 | **11.662286** | **17.850864** | **-25.52493** |
| LTT52.AVG_Signal | 3.915854 | 8.060923 | 57.2822 | LNT52.AVG_Signal | -3.45678 | 2.149506 | 48.12093 | **7.372637** | **5.911417** | **9.16127** |
| LTT30.AVG_Signal | -4.85394 | -3.83209 | 23.92586 | LNT30.AVG_Signal | -11.1136 | 4.435716 | 25.74211 | **6.25962** | **-8.267806** | **-1.81625** |
| LTT14.AVG_Signal | -2.17468 | 1.024604 | 58.75826 | LNT14.AVG_Signal | -8.29319 | -5.18411 | 22.50487 | **6.118511** | **6.208714** | **36.25339** |
| LTT12.AVG_Signal | -4.08411 | -5.55551 | 28.32432 | LNT12.AVG_Signal | -8.67137 | 20.59355 | 78.91606 | **4.587264** | **-26.149061** | **-50.59174** |
| LTT32.AVG_Signal | -9.65439 | -9.12344 | 26.34877 | LNT32.AVG_Signal | -11.6295 | 7.496103 | 28.68303 | **1.975096** | **-16.619541** | **-2.33426** |
| LTT38.AVG_Signal | -10.0189 | -5.39098 | 12.62063 | LNT38.AVG_Signal | -11.9559 | -7.28471 | 47.64776 | **1.93701** | **1.893724** | **-35.02713** |
| LTT11.AVG_Signal | -9.06064 | -22.1016 | 8.536842 | LNT11.AVG_Signal | -8.45045 | -5.42145 | 14.36031 | **-0.610183** | **-16.680171** | **-5.823468** |
| LTT47.AVG_Signal | -15.6546 | -6.26104 | 34.25648 | LNT47.AVG_Signal | -12.9174 | -4.24358 | 44.22153 | **-2.73722** | **-2.017469** | **-9.96505** |
| LTT49.AVG_Signal | -5.81031 | -11.9747 | -0.77139 | LNT49.AVG_Signal | -2.93039 | 8.748204 | 49.75808 | **-2.879923** | **-20.722904** | **-50.5294664** |
| LTT35.AVG_Signal | -8.67754 | -14.3213 | 14.6195 | LNT35.AVG_Signal | -5.49713 | 0.044276 | 40.05431 | **-3.180415** | **-14.3655363** | **-25.43481** |
| LTT08.AVG_Signal | -16.8128 | -23.8344 | 2.944532 | LNT08.AVG_Signal | -13.5998 | 4.467453 | 13.91087 | **-3.21305** | **-28.301873** | **-10.966338** |
| LTT13.AVG_Signal | -5.6263 | 74.05672 | 62.79569 | LNT13.AVG_Signal | -2.27967 | 25.73811 | 81.97675 | **-3.346629** | **48.31861** | **-19.18106** |
| LTT28.AVG_Signal | 2.821633 | 14.01068 | 43.5021 | LNT28.AVG_Signal | 6.931495 | -0.32416 | 16.84914 | **-4.109862** | **14.3348384** | **26.65296** |
| LTT25.AVG_Signal | -6.79553 | -2.07097 | 43.44896 | LNT25.AVG_Signal | -2.1399 | -4.23265 | 39.54658 | **-4.655632** | **2.161681** | **3.90238** |
| LTT33.AVG_Signal | -14.0728 | -9.61333 | 22.63374 | LNT33.AVG_Signal | -8.95594 | -10.1755 | 29.98331 | **-5.116866** | **0.562116** | **-7.34957** |
| LTT39.AVG_Signal | -16.1274 | -8.93955 | 16.88101 | LNT39.AVG_Signal | -10.9453 | -6.33886 | 16.74965 | **-5.18211** | **-2.600682** | **0.13136** |
| LTT05.AVG_Signal | -6.91459 | -14.8308 | 13.29479 | LNT05.AVG_Signal | -1.54209 | 0.891653 | 38.40672 | **-5.372503** | **-15.7224431** | **-25.11193** |
| LTT48.AVG_Signal | -4.50217 | -13.2405 | 28.52349 | LNT48.AVG_Signal | 1.213897 | 1.939086 | 31.44179 | **-5.716069** | **-15.179606** | **-2.9183** |
| LTT10.AVG_Signal | -7.38886 | -6.51957 | 34.8596 | LNT10.AVG_Signal | 3.663072 | 65.57564 | 198.5621 | **-11.051931** | **-72.095209** | **-163.7025** |
| LTT34.AVG_Signal | -12.8668 | -15.4301 | 36.75366 | LNT34.AVG_Signal | -0.32505 | 11.39186 | 58.39212 | **-12.5417857** | **-26.82193** | **-21.63846** |
| LTT36.AVG_Signal | -7.9502 | -7.80857 | 6.52828 | LNT36.AVG_Signal | 6.667596 | -0.19909 | 38.092 | **-14.617794** | **-7.6094786** | **-31.56372** |
| LTT29.AVG_Signal | -15.4419 | 4.399683 | 13.56411 | LNT29.AVG_Signal | -0.70113 | 2.548981 | 35.69946 | **-14.7407867** | **1.850702** | **-22.13535** |
| LTT03.AVG_Signal | -16.4018 | -13.1204 | 70.30713 | LNT03.AVG_Signal | -1.544 | -12.9034 | 30.19224 | **-14.857783** | **-0.21698** | **40.11489** |
| LTT50.AVG_Signal | -8.8576 | -5.46577 | 18.87685 | LNT50.AVG_Signal | 6.350345 | 24.10427 | 52.7311 | **-15.207949** | **-29.570036** | **-33.85425** |
| LTT21.AVG_Signal | -12.8892 | 8.581036 | 23.17283 | LNT21.AVG_Signal | 4.895322 | 24.0506 | 111.744 | **-17.784472** | **-15.469564** | **-88.57117** |
| LTT43.AVG_Signal | -11.5381 | -13.78 | 3.629772 | LNT43.AVG_Signal | 6.718066 | 7.111102 | 60.49253 | **-18.256206** | **-20.891132** | **-56.862758** |
| LTT19.AVG_Signal | -17.5878 | 12.21485 | 64.3429 | LNT19.AVG_Signal | 3.750607 | 21.59582 | 41.47648 | **-21.338357** | **-9.38097** | **22.86642** |
| LTT22.AVG_Signal | -4.12348 | 15.86934 | 35.23391 | LNT22.AVG_Signal | 17.3255 | 4.758834 | 42.58747 | **-21.448976** | **11.110506** | **-7.35356** |
| LTT27.AVG_Signal | -11.7997 | -13.3918 | 19.68449 | LNT27.AVG_Signal | 11.2783 | 4.757517 | 47.59362 | **-23.07804** | **-18.149337** | **-27.90913** |
| LTT17.AVG_Signal | -2.84323 | -1.48357 | 13.4833 | LNT17.AVG_Signal | 21.08902 | -9.36609 | 16.56286 | **-23.932254** | **7.882527** | **-3.07956** |
| LTT18.AVG_Signal | -12.0493 | -3.29477 | -1.00457 | LNT18.AVG_Signal | 19.79759 | 9.086266 | 33.62701 | **-31.84693** | **-12.381035** | **-34.63158** |
| LTT53.AVG_Signal | -15.9966 | -3.15452 | 87.84662 | LNT53.AVG_Signal | 33.00753 | -3.70379 | 22.40678 | **-49.0041** | **0.549266** | **65.43984** |
